# Learning under uncertainty—Conservation of populations and persistence of dynamic resources through adaptive switching feedback controllers

**DOI:** 10.1371/journal.pone.0349236

**Published:** 2026-06-01

**Authors:** Phoebe Smith, Chris Guiver

**Affiliations:** 1 Department of Mathematical Sciences, University of Bath, Bath, United Kingdom; 2 School of Computing, Engineering & the Built Environment, Edinburgh Napier University, Merchiston Campus, Edinburgh, United Kingdom; Karlstad University: Karlstads Universitet, SWEDEN

## Abstract

In the context of conservation under uncertainty, the problem of designing and analysing adaptive switching feedback control schemes for systems of positive difference equations is considered. The aim is to ensure persistence of the population, corresponding to the zero equilibrium of the closed-loop control system being unstable in a certain sense. A robust control approach is adopted, where multiple discrete control actions are available, corresponding to different management strategies or policies. However, the exact effect of each strategy is assumed to be uncertain. Based on principles from both positive dynamical systems and simple adaptive feedback control, a suite of control schemes is proposed from which a destabilising (persistent) strategy is selected based on a switching process, should such a strategy exist. We demonstrate that the switching rules can be augmented with several variations, altering the transient behaviour and thus tailoring them to the particular requirements of the user. The current work substantially builds upon and enhances earlier results of the authors, by establishing key and quite general hypotheses of the underlying model and control scheme to ensure persistence, so that the results are applicable to a wide range of model types. The proposed control schemes are illustrated with examples.

## Introduction

Achieving desirable outcomes through rational decision making is a challenge which arises in almost all scientific, social, and economic disciplines, including natural resource management and conservation. There is an understandably vast literature dedicated to the subject from a range of academic perspectives with monographs including, for instance, [1–4]. The successful conservation of managed resources or populations (see, from many possible works, for instance, [2]) has numerous overlapping (and often competing) dimensions, from ethical, economic, and viability, through to value judgments, arguably rendering it a wicked problem [5,6]. However, effective and urgent conservation action is needed across the globe, as the actions of humans threaten more species with widespread extinction now than ever before [7, Section A5].

The present work continues the line of enquiry of proposing and studying control-theoretic tools in ecology, here particularly in the context of conservation. The connections and overlap between control theory and biology/ecology are well known, with a range of results and perspectives appearing in, but not limited to, [8–15]. We refer the reader to [16] for an interesting discussion of the various meanings of the word “control” in a conservation context. Very roughly speaking, these connections exist and have proven highly fruitful as feedback, uncertainty, and the desire to both understand and often control dynamic behaviour are intrinsic to both disciplines. As we shall describe, we use a *positive systems model-based* and *robust adaptive control* approach, as we assume that significant uncertainty is present.

Control engineering, and the underlying mathematical field of control theory, offer significant bodies of knowledge of how to keep the dynamic behaviour of processes within an acceptable range even if the underlying model is based on uncertainties or the real processes cannot be mapped and modelled completely [17]. These disciplines trace their roots back to the industrial revolution and the robust regulation of electrical and mechanical systems, with modern treatments including, for instance, [18–21]. Control systems and feedback loops are ubiquitous in science and engineering, and arise in traditional areas from aerospace control, manufacturing and process control, through to robotics, electrical power systems, systems biology, and therapeutics. Their study and subsequent use have been fantastically successful [22]. Novel and emerging applications where control systems play an essential role range from the control of autonomous vehicles, smart grids and devices, through to epidemiology [23]. In fact, so pervasive is control engineering across science and technology that it has been called *the hidden technology* [24]. Writing in 2026, there is renewed interest in the intersection of control theory and machine learning, particularly seeking to integrate formal control-theoretic guarantees of robustness or performance into large, high-dimensional, data-driven dynamical systems [25–27].

Positive systems are dynamical systems where the evolution map leaves a positive cone invariant. They are a logical choice for modelling populations, which are naturally temporally-varying nonnegative quantities. Positive systems have been studied extensively owing to their rich mathematical structure as well their relevance in a wide range of contexts, from economics and logistics, through to pharmacokinetics and telecommunications (see, for instance, [28, Chapter 1]), with key texts [29,30]. The inclusion of control terms in positive dynamical systems leads to so-called positive control systems [28,31,32], with recent review papers [33,34]. From a technical, mathematical perspective, the arguably obvious goal of many conservation applications is to guarantee persistence of populations; roughly speaking meaning that solutions avoid, or are repelled from, the zero equilibrium, which corresponds to population absence. More precisely, to quote Smith & Thieme [35, p.ix]: “*Persistence theory can give a mathematically rigorous answer to the question of persistence by establishing a positive lower bound for the long-term value of a component of a dynamical system*.” Persistence theory is an accepted and important tool in the study of theoretical population dynamics, as evidenced across, for example, the works [36–44].

Robust control is an approach to control theory which, in broad terms, seeks to ensure that control algorithms are robust to uncertainties and disturbances, in other words, meaning that they perform as intended in the presence of such uncertainties [18,45–47]. For example, in the context of modelling populations, where models are often crude and data limited, uncertainty is plentiful and arises through choice of model structure, parametric uncertainty, and unknown disturbances to the dynamics [48,49]. The history of modern robust control dates back to 1970s and the realisation of control engineers that over-optimisation in the presence of uncertainty leads to fragility [50]. For recent studies on robust control we refer to, for example, the works of Bounemeur & Chemachema [51–54]. Adaptive control is one strand of robust control, and is a broad term with no single agreed definition. However, a key and common theme across adaptive control is that a control variable is determined dynamically (adaptively) as a function of a measured variable, in such a way that renders the uncertain closed-loop feedback system stable in some sense. For further information on the theory and development of adaptive control we direct the reader to, for example, the papers: [55–58], as well as [59] for historical notes. As noted in [49], the term “adaptive” in a control-theoretic setting refers to one of the particular feedback control frameworks mentioned above, whilst in the resource and ecological management field of adaptive management (see, for instance, [60–62]), adaptive typically refers to the use of a feedback to incorporate learning into management. In that sense, there are both parallels and differences between the use of the term “adaptive” in these two contexts.

In this paper, we are inspired by adaptive control theory as a tool to aid the management of populations that are of conservation interest, yet are subject to considerable uncertainty. We propose a so-called adaptive switching feedback control scheme as an instrument to aid such conservation efforts. In overview, the scheme uses feedback to select a control strategy (corresponding to what might be termed a management action or policy intervention) from a given and fixed set of available control strategies, to ensure persistence of a population, should such a control strategy be present. Crucial to our development is the realistic assumption that the overall effect of each control strategy is unknown. Of course, in the case that the effects of each control strategy *are* known then, all else being equal, a population manager or practitioner would clearly choose an appropriate strategy which gives the desired dynamic behaviour. However, many conservation tactics affect the population of interest indirectly. For instance, planting more trees to combat deforestation and habitat fragmentation will influence the population growth of reliant species. In cases like this, the exact quantitative effect of the control action may not be clear, ergo the overall effects of the control strategies available are likely to be subject to substantial uncertainty.

The starting point for a mathematical description of the problem is the following system of nonlinear, discrete-time difference equations


$$\begin{array}{c} x(t+1)=F(h,x(t)),\;x(0)=x_{0},\;t\in Z_{+}:=\{0,1,2,\ldots \}. \end{array}$$ (1)

Here, $x(t)\in R_{+}^{n}$ denotes a population of interest at discrete time-step *t*, and may be scalar- or vector-valued, corresponding to *n* = 1 and *n* > 1, respectively, *t*he latter representing structured populations [63,64]. The term *x*_0_ denotes the initial population. We call *x* the state variable. The integer *h* in Eq (1) determines which of the *q* available control strategies is applied, and the function F(h,·) describes the dynamics of *x* under control strategy *h*. It is assumed that *F*(*h*,0) = 0 for all *h*, thus *x* = 0 is a constant (equilibrium) solution of Eq (1), corresponding to population absence.

The adaptive switching feedback control scheme updates *x* during each time step using the following switched system


$$\begin{array}{c} x(t+1)=F(𝒦(s(t)),x(t)),\;x(0)=x_{0},\;t\in Z_{+}, \end{array}$$ (2)

where 𝒦(z) is an integer between 1 and *q* for all nonnegative real *z*, indicating which control strategy is applied at time *t*. The variable *s* is the so-called switching sequence, a choice of the designer, and is *t*o be determined as a function of a measured variable, itself typically some portion of the whole state *x*(*t*).

Here we present a suite of update laws for the switching sequence *s* in Eq (2) which ensure population persistence of the resulting closed-loop feedback system by identifying and converging to a “persistent strategy”, should one exist. The adaptive switching feedback controllers considered only impose structural (and rather mild) hypotheses on the models F(h,·), and the measured variable, and so possess considerable robustness properties which we describe in detail. In summary, the adaptive switching feedback control scheme learns a persistent control strategy under potentially considerable uncertainty. Our main results are a suite of theoretical and numerical findings for Eq (2) encompassing both: (i) a range of uncertain population models F(h,·); and, (ii) a choice of design of switching sequence. Technically, our work crucially draws upon the assumed positive dynamical systems structure, including persistence concepts, as well as ideas from the field of adaptive control theory.

### Contributions

In terms of overall contribution, the work continues to demonstrate the potential utility of tools from control theory and positive dynamical systems in theoretical ecology scenarios, presently focussing on novel robust control techniques which are well-suited for scenarios where large uncertainty is to be expected. More specifically:

- There is arguably a research gap in the development of highly-robust control-theoretic tools for the timely and important societal challenges of conservation or dynamic resource management, with a few notable exceptions which we consider in the Discussion. Real-world conservation efforts are laborious and expensive, and so there is value in undertaking comparatively inexpensive prior theoretical and computational research. Thus, our current work continues to fill this gap. The main comparable work the authors are aware of is our earlier paper [65] which, to the best of the authors’ knowledge, first considered adaptive control in the context of conservation. Our present results substantially improve those of [65], briefly, by the wider range of models to which the theory developed applies, as well as the innovations of the adaptive switching feedback control schemes presented.
- The current work complements the papers [49,66,67], which all use adaptive control theory to address stabilisation problems (of the zero equilibrium) of positive systems, in the context of managing pest or invasive species. Indeed, a key difference between the current work and these papers is that here the adaptive switching feedback control schemes seek to *destabilise* the zero equilibrium and ensure population persistence, rather than stabilise the zero equilibrium. Therefore, the overlap of these papers and the present work is minimal.
- Our work provides new technical insight and understanding of key theoretical ingredients required for adaptive control tools to successfully apply to positive systems, and particularly explores connections between adaptive control and persistence properties. Briefly, our present work places assumptions on the type of dynamic behaviour the model is required to have (which is mild and ecologically realistic, such as all solutions being bounded), rather than specifying the *type* of model which generates the behaviour, say, in terms of its structure or parameters.
- Practically, the variations we propose to the adaptive switching feedback control scheme demonstrate how the schemes can be adjusted in line with the users needs, and to bespoke settings. In essence, the variations either use more information available to alter (and ideally improve) transient behaviour, or are better suited to accommodating oscillatory populations. These variations further distinguish the present work from [49,65–67], yet the variations we propose may be applicable in these settings as well. Moreover, we demonstrate how the results are robust to various forms of uncertainty, including measurement delays and certain actuator malfunctions, which are important and meaningful contributions for the intended application of conservation.
- Finally, whilst the main motivation for the current study is to applications in conservation under uncertainty, the results are applicable to positive control systems more generally.

### Paper organisation

The remainder of the work is organised as follows. After recording required notation, we provide our main theoretical results on the adaptive switching feedback control scheme for Eq (1) and present a number of variations that, in overview, alter the transient behaviour of the scheme. We then perform numerical simulations for several worked examples. Finally, we provide a discussion and summarise our results. In S1 Appendix, we provide technical details not given in the main text, as well as supporting information for the examples.

### Notation

Most mathematical notation we use is standard, and so only a few items are mentioned. The symbols $Z_{+}$ and N denote the nonnegative and positive integers, respectively. For positive integer *q* we write $\underset{―}{q}=\{1,2,\ldots ,q\}$ for brevity. We let $R^{n}$ denote usual *n*-dimensional Euclidean space, for positive integer *n*, and $R_{+}^{n}$ denotes the nonnegative orthant of componentwise nonnegative vectors. Similarly, we let $R^{m\times n}$ and $R_{+}^{m\times n}$ denote the sets of size *m* × *n* matrices, and the subset of componentwise nonnegative matrices, respectively. For $z\in R^{n}$, we write *z* ≥ 0 if $z\in R_{+}^{n}$, and z≫0 if every component of *z* is positive. We use the same symbols for componentwise nonnegativity and positivity of matrices, respectively.

The symbol $\|\cdot {\|}_{1}$ denotes the Euclidean one-norm, which is a natural choice in the context of population modelling, as it corresponds to the total number (or density) of individuals. In the sequel we let ‖·‖ denote any monotonic norm on $R^{n}$, that is, for all $z_{1},z_{2}\in R^{n}$ it follows that $\left|z_{1}|\leq |z_{2}\right|$ implies that $\left\|z_{1}\|\leq \|z_{2}\right\|$. Here $|z|\in R^{n}$ for $z\in R^{n}$ and $\left|z{|}_{i}=|z_{i}\right|$ for i=1,2,…,n (vector of componentwise absolute values).

For square matrix $A\in R^{n\times n}$, we let ρ(A) denote the spectral radius of *A*.

## An adaptive switching feedback control scheme

We begin by introducing the adaptive switching feedback control scheme, before stating and proving our main theoretical results. Finally, we propose variations to the scheme which take advantage of more of the data available to population managers.

### Switching preliminaries

Throughout the work it is assumed that there are *q* > 1 available control strategies, and that each strategy gives rise to a population model, captured by F(h,·) for $h\in \underset{―}{q}=\{1,2,\ldots ,q\}$. For brevity from hereon in, we use the term “strategy” for “control strategy” throughout. Each F(h,·) is a function $R_{+}^{n}\to R_{+}^{n}$, ensuring that *x*(*t*) ≥ 0 for all $t\in Z_{+}$ whenever *x*(0) ≥ 0 — natural positivity assumptions for ecologically meaningful models.

Later, *q*_e_ and *q*_p_ shall denote a partition of $\underset{―}{q}$ of indices of strategies which lead to population extinction and persistence, respectively, so that


$$\begin{array}{c} q_{e}\cap q_{p}=\emptyset \;\text{and}\;q_{e}\cup q_{p}=\underset{―}{q}. \end{array}$$ (3)

We emphasise that it is assumed that the population managers *do not* know which strategies correspond to persistence or extinction, otherwise they would not require the assistance of the adaptive switching feedback control scheme.

Another key premise of the work is that the whole state *x* is not necessarily known or available. Rather, there is a known measured variable, denoted *y*, given by


$$\begin{array}{c} y(t)=\eta (x(t)), \end{array}$$ (4)

where $\eta :R_{+}^{n}\to R_{+}^{p}$ is given, for positive integer *p*, corresponding to the number of measurements of *x* that are recorded per time step. Note that η may transform *y*(*t*) such that it is not the same dimension as *x*(*t*), corresponding to *n* ≠ *p*.

The second ingredient is a sequence τ which is constructed thus:

**(T)** τ is a positive, strictly increasing and unbounded (scalar) sequence with τ(0)=0 and such that


$$\begin{array}{c} \frac{\tau (j+1)}{\tau (j)}\to \infty \;\text{as}\;j\to \infty . \end{array}$$

The growth property of τ means that τ grows faster than exponentially for any given exponent. As an example, the sequence τ defined by


$$\begin{array}{c} \tau (k+1)=1+(k+1)\tau (k),\;k\in Z_{+},\;\tau (0)=0. \end{array}$$

satisfies assumption **(T)**. With the sequence τ as above, the function $𝒦:R_{+}\to \underset{―}{q}$ is defined by


$$\begin{array}{c} 𝒦(z):=\left\{ \begin{array}{cccc} & 1, & z & =0,\\ & (j\;\;\mathrm{mod}\;q)+1, & z & \in (\tau (j-1),\tau (j)],\;j\in N, \end{array}\right.\;\forall \;z\in R_{+}. \end{array}$$ (5)

In words, for any given *z* ≥ 0, the evaluation 𝒦(z) returns an integer $h\in \underset{―}{q}$, which shall be used to determine which strategy is applied. The use of modular arithmetic yields that strategies are cycled through consecutively as *z* increases through intervals of the form (τ(j−1),τ(j)] for integer *j*. For convenience, we call such intervals τ *intervals*.

The function 𝒦 in Eq (2) is evaluated using the *switching sequence*, denoted by *s*, which itself is a dynamic variable determined as a function of the measured variable *y*. We call this function the *switching sequence update law*, denoted by $r:R_{+}\to R_{+}$, and proceed to discuss its construction. For decreasing function $\chi :(0,\infty )\to R_{+}$ and *M* > 0, we define *r* by:


$$\begin{array}{c} r_{\chi }(z\;;M):=\left\{ \begin{array}{cccc} & 0, & M\leq z & ,\;z=0,\\ & \chi (z), & 0<z & <M. \end{array}\right. \end{array}$$ (6)

Since no confusion is likely, we write *r* for $r_{\chi }$. Then, the switching sequence *s* is updated via


$$\begin{array}{cc} s(t+1) & =s(t)+r(\|y(t)\|\;;M)\\ & =s(t)+\left\{ \begin{array}{ccc} & 0, & M\leq \|y(t)\|,\|y(t)\|=0,\\ & \chi (\|y(t)\|), & 0<\|y(t)\|<M, \end{array}\right.\;s(0)=s_{0}. \end{array}$$ (7)

In words, the positive constant *M*, called the *switching threshold*, denotes the desired level of population persistence and ‖y(t)‖<M corresponds to being in an undesirable position. In this case, *s*(*t*) increases by χ(‖y(t)‖) at each time-step — increments which increase as ‖y(t)‖ decreases by choice of χ. The function χ plays the role of determining how *s* increases as a function of the measured variable. As the value of *s*(*t*) increases, it moves further along the current τ interval, and a switch of strategy occurs when *s* enters a larger τ interval. Of course, the aim is to switch out of strategies which are undesirable, and switch into ones which are desirable. The switching sequence does not increase when ‖y(t)‖≥M. The initial switching sequence value *s*_0_, the switching threshold *M*, and the function χ are design parameters.

**Fig 1:**
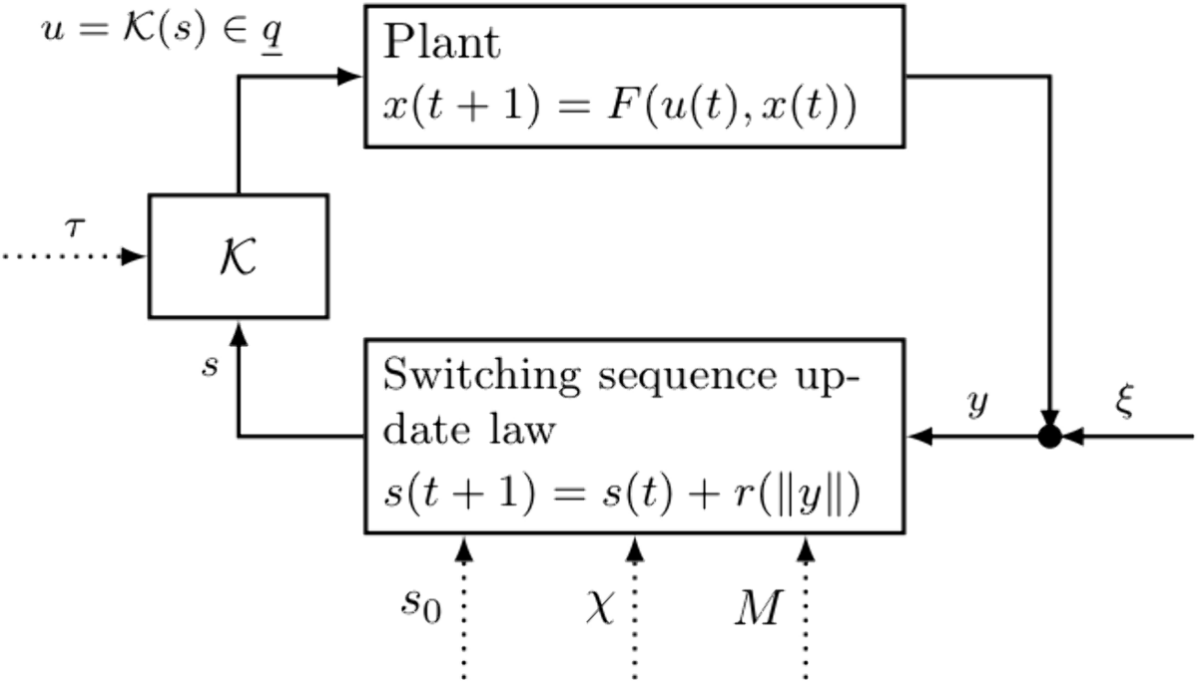
Block diagram of the adaptive switching feedback control scheme in Eq (8). Dotted lines denote required design parameters. The term ξ denotes measurement noise, which is equal to zero (absent) in Eq (8), but appears in later scenarios.

The feedback connection of Eq (2) (with measurement Eq (4)) and Eq (7) gives rise to the adaptive switching feedback control scheme


$$\begin{array}{c} \begin{array}{c} \begin{array}{ccc} x(t+1) & =F(𝒦(s(t)),x(t)),\; & x(0)=x_{0},\\ y(t) & =\eta (x(t)),\\ s(t+1) & =s(t)+r(\|y(t)\|\;;M),\; & s(0)=s_{0}, \end{array}\}\;t\in Z_{+}, \end{array} \end{array}$$ (8)

with unique solution denoted (*x*,*s*). Fig 1 contains a block diagram of the adaptive switching feedback control scheme in Eq (8).

**Remark 1.** Our earlier work [65, Equation (2.1)] made extensive use of the switching sequence


$$\begin{array}{c} s(t+1)=s(t)+\left\{ \begin{array}{cccc} & 0, & M\leq \|y(t){\|}_{1} & ,\;\|y(t){\|}_{1}=0,\\ & \frac{1}{\|y(t){\|}_{1}}, & 0<\|y(t){\|}_{1} & <M, \end{array}\right.\;s(0)=s_{0},\;t\in Z_{+}, \end{array}$$ (9)

which, we note, is a special case of Eq (7) with χ(z)=1/z and $\|\cdot \|=\|\cdot {\|}_{1}$.

### A persistence concept

Here we outline a required persistence (also termed persistency) concept. We propose one somewhat inspired by that in [39, Section 4]. Namely, for fixed *R* > 0, we say that Eq (1) is *uniformly ultimately semi-globally* η*-persistent* if there exists ε>0 such that, for every compact set $\Gamma \subseteq R_{+}^{n}$ with 0∉Γ and bounded by *R*, there exists $\ell \in Z_{+}$ such that


$$\begin{array}{c} \|y(t+\ell )\|=||\eta (x(t+\ell ;x(0),h))||\geq \varepsilon \;\forall \;x(0)\in \Gamma ,\;\forall \;t\in Z_{+}. \end{array}$$ (10)

The key properties of the above are that there is some positive ε which acts as a lower bound for ‖y(t)‖=‖η(x(t))‖ for all non-zero *x*(0) (hence uniform) and sufficiently large *t* (hence ultimate). Moreover, the time taken to reach this bound is semi-global in that, for each compact set of initial states Γ bounded by *R*, the same persistence time ℓ “works” for all x(0)∈Γ. The constant *R* can be arbitrarily large, but is fixed. In principle, the above quantities ε and ℓ may depend on *R*, but it will not play a large role. It is included to simplify some of the technical arguments, and its inclusion is certainly not a practical concern. For convenience we call ε in Eq (10) a *persistence threshold*, and we shall refer to persistence (in the above sense), or not, as a property of the strategy $h\in \underset{―}{q}$ in Eq (1).

We require a further technical refinement of the above persistence property. Fix 0<δ<Δ, γ∈(0,1) and write


$$\begin{array}{c} \Gamma_{\gamma }(t):=\{z\in R_{+}^{n}\;:\;\delta \gamma^{t}\leq \|z\|\leq \Delta \}. \end{array}$$ (11)

Set $\ell_{t}=\ell (\Gamma_{\gamma }(t))$, where ℓ is as in Eq (10). We say that $\ell_{t}$ has *affine-linear growth* if, for every 0<δ<Δ and γ∈(0,1), there exist constants *a*,*b* > 0 such that $\ell_{t}=\ell (\Gamma_{\gamma }(t))$ satisfies


$$\begin{array}{c} \ell_{t}\leq a+tb\;\forall \;t\in Z_{+}. \end{array}$$ (12)

We also introduce a new partition of the *q* available strategies; it allows strategies which are persistent below a desirable level to be classed as undesirable strategies. We use *q*_d_ and *q*_u_ to denote desirable and undesirable strategies, respectively, such that


$$\begin{array}{c} q_{d}\subseteq q_{p}\;\text{and}\;q_{u}=q_{e}\cup (q_{p}⧵q_{d})\;\text{so that}\;q_{u}\cap q_{d}=\emptyset \;\text{and}\;q_{u}\cup q_{d}=\underset{―}{q}, \end{array}$$

where *q*_e_ and *q*_p_ are as in Eq (3). We note that a strategy which gives rise to $\|y(t_{k})\|<M$ periodically along some subsequence $(t_{k}{)}_{k}$ is deemed an undesirable strategy.

### Model hypotheses

Our main theoretical results require the following hypotheses, stated next and with commentary below:

**(H1)** For every bounded $\Gamma \subseteq R_{+}^{n}$ the solution *x* of


$$\begin{array}{c} x(t+1)=F(v(t),x(t)),\;t\in Z_{+},\;x(0)\in \Gamma , \end{array}$$ (13)

is bounded, independently of $v:Z_{+}\to \underset{―}{q}$.

**(H2)** For every bounded $\Gamma \subseteq R_{+}^{n}$, there exist λ>0, γ∈(0,1) such that the solution *x* of Eq (13) satisfies


$$\begin{array}{c} \|x(t_{1}+t_{2})\|\geq \lambda \gamma^{t_{1}}\|x(t_{2})\|\;\forall \;t_{1},t_{2}\in Z_{+}, \end{array}$$

independently of $v:Z_{+}\to \underset{―}{q}$.

**(H3)** There exists at least one $h\in \underset{―}{q}$ that is uniformly ultimately semi-globally η-persistent with corresponding ℓ as in Eq (10) satisfying the affine-linear growth property.

**(H4)** For every bounded $\Gamma \subseteq R_{+}^{n}$, there exist $k,m\in Z_{+}$ with *m* ≤ *k*, and a constant *c* > 0 such that the solution *x* of Eq (13) satisfies


$$\begin{array}{c} c\|x(t-m)\|\leq \|y(t)\|\;\forall \;t\in Z_{+},\;t\geq k, \end{array}$$

independently of $v:Z_{+}\to \underset{―}{q}$.

**(H5)** The function χ respects exponential growth in the sense that, for all *d*_1_, *d*_2_ > 0, there exist *d*_3_ > 0 and *d*_4_ > 1 such that


$$\begin{array}{c} \chi (d_{1}d_{2}^{t})\leq d_{3}d_{4}^{t}\;\forall \;t\in Z_{+}. \end{array}$$

Some remarks on the above hypotheses are in order, after which we provide a technical result containing sufficient conditions under which they are valid, and demonstrate how they apply in a concrete example. In overview, hypotheses **(H1)**– **(H3)** relate to the underlying to-be-controlled models in Eqs (1) and (8), captured by the functions *F*. Hypothesis **(H4)** is a coupling condition between the states and measured variables, and hypothesis **(H5)** is a technical requirement of the switching sequence *s* in Eq (7). Hypothesis **(H1)** is a mild boundedness requirement, and ensures that the *x* component of solutions (*x*,*s*) of Eq (8) is bounded, uniformly on bounded sets of conditions for *x* and independently of the switching sequence. We note that **(H1)** rules out the situation wherein F(h,·) equals the linear unstable model *F*(*h*, *z*) = *A*_*h*_*z* with $\rho (A_{h})>1$, but this situation is arguably not biologically realistic, and was a focus of [65], so is not considered again here. In words, hypothesis **(H2)** states that the *x* component of solutions (*x*,*s*) of Eq (8) decays at fastest exponentially, uniformly on bounded sets of initial conditions for *x*, and independently of the switching sequence.

Hypothesis **(H3)** is the key persistence ingredient, and persistent in the sense we have described. To motivate the introduced affine-linear growth property, note that as *t* increases, the set $\Gamma_{\gamma }(t)$ in Eq (11) contains non-zero elements converging exponentially to zero. It is intuitively to be expected that the measured variable *y* corresponding to such initial states in $\Gamma_{\gamma }(t)$ takes longer for Eq (10) to hold, captured by increasing $\ell =\ell_{t}$. Thus, the affine-linear growth property Eq (12) bounds how fast $\ell_{t}$ can grow as a function of *t*.

We highlight that hypotheses **(H1)**– **(H3)** are qualitative, and are deliberately formulated to be widely applicable. For instance, they are independent of exact knowledge of specific model structure or parameter values, and hence are robust with respect to model- or parametric-uncertainty. We argue that this is likely to be important in a conservation context as there is often little time to improve the knowledge of the system before action is required to stop the population declining further [68]. Furthermore, **(H1)**– **(H3)** capture realistic properties of the ecological models primarily under consideration, and hence are arguably reasonable assumptions.

The coupling condition between states and outputs **(H4)** basically requires that, possibly after some transient time *k*, the norm of the measured variable y=η(x) is an upper bound for the norm of the state *x*, up to some multiplicative constant and possibly with some delay *m* in *x*. Again, this bound is required to hold independently of the switching sequence. The arguably simplest case wherein **(H4)** holds is when the output equals the state, that is, *y* = *x*, by taking *m* = *k* = 0 and *c* = 1. Similarly, in the case that the population abundance is measured, that is, y=η(x)=‖x‖, then hypothesis **(H4)** holds once **(H2)** does.

Finally, hypothesis **(H5)** is technical, and requires that the function χ in Eqs (7) and (8) with argument equal to an exponential term grows no faster than exponentially. Recall that since χ is chosen by the user, this requirement can always be fulfilled by construction. Hypothesis **(H5)** holds if χ decreases polynomially, whilst the function χ given by χ(z)=exp(1/z) is an example for which it fails.

The following lemma contains sufficient conditions for hypotheses **(H1)**– **(H4)**. The proof essentially leverages comparison arguments which are inherent to positive dynamical systems. It is elementary, but lengthy, and so to avoid disruption to the present flow of ideas, the proof is relegated to Section 1 in S1 Appendix.

**Lemma 2.** *Consider the adaptive switching feedback control scheme in Eq (8), and assume that η is non-decreasing. The following statements hold.*

(i) *If there exist* $X_{1}\in R_{+}^{n\times n}$ *with* $\rho (X_{1})<1$ *and* $X_{2}\in R_{+}^{n}$ *such that*
  $$\begin{array}{c} F(h,z)\leq X_{1}z+X_{2}\;\forall \;z\in R_{+}^{n},\;\forall \;h\in \underset{―}{q}, \end{array}$$ (14)
  *then* **(H1)** *holds.*
(ii) *If* **(H1)** *holds and, for all* γ>0*, there exists irreducible* $A_{\gamma }\in R_{+}^{n\times n}$ *such that*
  $$\begin{array}{c} F(h,z)\geq A_{\gamma }z\;\forall \;z\in R_{+}^{n},\;\|z\|\leq \gamma ,\;\forall \;h\in \underset{―}{q}, \end{array}$$ (15)
  *then* **(H2)** *holds.*
(iii) *If* **(H1)** *and* Eq (15) *hold and, for all* γ>0*, the matrix* $A_{\gamma }$ *also satisfies*
  $$\begin{array}{c} \|\eta (A_{\gamma }^{\nu }z)\|\geq c\|z\|\;\forall \;z\in R_{+}^{n},\;\|z\|\leq \gamma , \end{array}$$ (16)
  *for some* $\nu \in Z_{+}$ *and c > 0, then* **(H4)** *holds.*
(iv) *If* Eqs (14)–(16) *are satisfied, and there exist* $\gamma_{1}>0$, $A_{\gamma_{1}}\in R_{+}^{n\times n}$ *with* $\rho (A_{\gamma_{1}})>1$ *and* $h\in \underset{―}{q}$ *such that*
  $$\begin{array}{c} F(h,z)\geq A_{\gamma_{1}}z\;\forall \;z\in R_{+}^{n},\;\|z\|\leq \gamma_{1}, \end{array}$$ (17)
  *then* **(H3)** *holds.*

By way of terminology, the function $\eta :R_{+}^{n}\to R_{+}^{p}$ is said to be *non-decreasing* if, for all $z_{1},z_{2}\in R_{+}^{n}$, it follows that


$$\begin{array}{c} z_{1}\leq z_{2}\;\Rightarrow \;\eta (z_{1})\leq \eta (z_{2}), \end{array}$$

which is a sensible property of measurement functions for populations, and so is not restrictive. Recall that the square matrix $M\in R^{n\times n}$ with components *m*_*ij*_ is said to be *reducible* if there exist non-empty disjoint subsets $J_{1},J_{2}\subseteq \underset{―}{n}$ such that $J_{1}\cup J_{2}=\underset{―}{n}$ and *m*_*ij*_ = 0 for all $(i,j)\in J_{1}\times J_{2}$. The matrix *M* is *irreducible* if it is not reducible. Irreducibility is a natural assumption for meaningful ecological models, see [69], and mathematically important as a suitable version of the Perron-Frobenius Theorem applies to irreducible matrices (see, for instance, [29, Theorem 1.4, p.27]).

**Example 3** (Verifying hypotheses **(H1)**– **(H5)** for the Allen-Clark model). We consider Eq (1) where the underlying model is a so-called Allen-Clark model. These objects are named after Allen [70] and Clark [71], see also [72] for more historical information, and are also a form of so-called Lur’e system with a time delay; see [41]. Delays are typically included in discrete-time population models when recruitment takes place several time steps after birth, meaning that a first-order difference equation is not an adequate representation of the real-world dynamics of a population [73]. In situations such as these, the inclusion of delays in difference equations leads to so-called higher-order difference equations.

For each strategy $h\in \underset{―}{q}$, the Allen-Clark models presently under consideration are given by


$$\begin{array}{c} z(t+1)=\alpha_{h}z(t)+\beta_{h}g(z(t-k);u_{h},\kappa_{h}),\;\forall \;t\in Z_{+},\;z(-j)=z_{-j}. \end{array}$$ (18)

Here: $z(t)\in R_{+}$ is the (scalar) population size at time *t*; $k\in Z_{+}$ is the time delay; $\alpha_{h}\in (0,1)$ and $\beta_{h}>0$ are positive scalars, and; the function *g* captures recruitment, and depends on the parameters $u_{h}$and $\kappa_{h}$. The parameters $\alpha_{h},\beta_{h},u_{h}$ and $\kappa_{h}$ are all assumed to depend on the strategy *h*. The *z*_−*j*_ terms for j=0,1,…,k are initial conditions. We comment that, within the literature (e.g., [73,74]), Allen-Clark models are often given in the form


$$\begin{array}{c} z(t+1)=\alpha z(t)+(1-\alpha )g(z(t-k)),\;t\geq 0, \end{array}$$

for 0<α<1, which is a special case of Eq (18) with *q* = 1.

Model Eq (18) can be expressed in the form Eq (1) by introducing the augmented state vector


$$\begin{array}{c} x(t)=(\begin{array}{c} z(t)\\ z(t-1)\\ z(t-2)\\ \vdots \\ z(t-k) \end{array})\in R_{+}^{k+1}=R_{+}^{n},\;\forall \;t\in Z_{+}, \end{array}$$

(*n*: = *k* + 1) which leads to


$$\begin{array}{c} x(t+1)=A_{h}x(t)+b_{h}g(c^{⊤}x(t);u_{h},\kappa_{h})=F(h,x(t)),\;\forall \;t\in Z_{+}, \end{array}$$ (19)

where $A_{h}\in R_{+}^{n\times n}$ and $b_{h},c\in R_{+}^{n}$ are given by


$$\begin{array}{c} A_{h}=(\begin{array}{ccccc} \alpha_{h} & 0 & \ldots & \ldots & 0\\ 1 & 0 & \ldots & \ldots & 0\\ 0 & 1 & \ddots & & \vdots \\ \vdots & \ddots & \ddots & \ddots & \vdots \\ 0 & \ldots & 0 & 1 & 0 \end{array}),\;b_{h}=(\begin{array}{c} \beta_{h}\\ 0\\ \vdots \\ 0 \end{array}),\;\text{and}\;c^{⊤}=(\begin{array}{cccc} 0 & \ldots & 0 & 1 \end{array}). \end{array}$$

We assume that, for all $h\in \underset{―}{q}$, the functions $z\mapsto g(z;u_{h},\kappa_{h})$ are continuous with $g(0;u_{h},\kappa_{h})=0$, and $g(z;u_{h},\kappa_{h})>0$ for *z* > 0. Moreover, we assume that the function


$$\begin{array}{c} z\mapsto \mu (z):=\max_{h\in \underset{―}{q}}g(z;u_{h},\kappa_{h}) \end{array}$$ (20)

is bounded. These assumptions are all ecologically reasonable. The last ingredient of the model is the measured variable. We assume that


$$\begin{array}{c} y(t)=z(t)=f^{⊤}x(t)=(\begin{array}{cccc} 1 & 0 & \ldots & 0 \end{array})x(t),\;t\in Z_{+}, \end{array}$$ (21)

that is, $\eta (z)=f^{⊤}z$. Clearly, the function η is non-decreasing.

We claim that hypotheses **(H1)**– **(H4)** hold, which we verify by invoking Lemma 2. For this purpose, observe that from the structure of the *A*_*h*_ and *b*_*h*_ it follows that, for all $h\in \underset{―}{q}$,


$$\begin{array}{c} \min_{m\in \underset{―}{q}}A_{m}=:A_{min}\leq A_{h}\leq A_{max}:=\max_{m\in \underset{―}{q}}A_{m}\\ \text{and}\;\min_{m\in \underset{―}{q}}b_{m}=:b_{min}\leq b_{h}\leq b_{max}:=\max_{m\in \underset{―}{q}}b_{m}, \end{array}$$

and $\rho (A_{max})=\max_{h\in \underset{―}{q}}\alpha_{h}<1$. Let $\mu_{max}$ denote an upper bound for μ in Eq (20). Invoking Eq (19), we estimate that


$$\begin{array}{c} F(h,z)=A_{h}z+b_{h}g(c^{⊤}z;u_{h},\kappa_{h})\leq A_{max}z+\mu_{max}b_{max},\;\forall \;z\in R_{+}^{n},\;\forall \;h\in \underset{―}{q}, \end{array}$$

so that **(H1)** holds by statement (i) of Lemma 2.

To verify **(H2)**, let γ>0 be given. By continuity and positivity of *g*, it follows that there exists δ>0 such that


$$\begin{array}{c} \min_{h\in \underset{―}{q}}g(z;u_{h},\kappa_{h})\geq \delta z,\;\forall \;z\in [0,\|c^{⊤}\|\gamma ]. \end{array}$$ (22)

Therefore, for $z\in R_{+}^{n}$ with ‖z‖≤γ, we have that $\|c^{⊤}z\|\leq \|c^{⊤}\|\gamma$. Consequently, by Eq (22)


$$\begin{array}{c} \begin{array}{cc} F(h,z) & \;\;\;=A_{h}z+b_{h}g(c^{⊤}z;u_{h},\kappa_{h})\geq A_{min}z+\delta b_{min}c^{⊤}z\\ & \;\;\;=(A_{min}+\delta b_{min}c^{⊤})z,\;\forall \;h\in \underset{―}{q}. \end{array} \end{array}$$

We compute that


$$\begin{array}{c} {𝒜}_{\delta }=A_{min}+\delta b_{min}c^{⊤}=(\begin{array}{ccccc} \alpha_{min} & 0 & \ldots & \ldots & \delta \beta_{min}\\ 1 & 0 & \ldots & \ldots & 0\\ 0 & 1 & \ddots & & \vdots \\ \vdots & \ddots & \ddots & \ddots & \vdots \\ 0 & \ldots & 0 & 1 & 0 \end{array}), \end{array}$$

is irreducible. We conclude that **(H2)** holds by statement (ii) of Lemma 2.

Furthermore, the matrix ${𝒜}_{\delta }$ is irreducible with positive trace and hence primitive (that is, there exists ν∈N such that ${𝒜}_{\delta }^{\nu }\gg 0$) by, for example, [75, Example 8.3.3]. Therefore, it follows that $f^{⊤}{𝒜}_{\delta }^{\nu }\gg 0$, and so


$$\begin{array}{c} \|\eta ({𝒜}_{\delta }^{\nu }z)\|=\|f^{⊤}{𝒜}_{\delta }^{\nu }z\|\geq c_{0}\|z\|,\;\forall \;z\in R_{+}^{n}, \end{array}$$

for suitable positive constant *c*_0_. Consequently, Eq (16) holds, and hence so does **(H4)** by statement (iii) of Lemma 2.

For property **(H3)**, from the theory of stability radii for positive systems, and particularly [76, Lemma 3.2], we have that


$$\begin{array}{c} \rho (A_{h}+\delta b_{h}c^{⊤})>1\;\text{if, and only if, }\;\delta >\frac{1}{c^{⊤}(I-A_{h}{)}^{-1}b_{h}}=\frac{1-\alpha_{h}}{\beta_{h}}, \end{array}$$

the final equality following from an elementary calculation. In particular, if there exist $h\in \underset{―}{q}$, $\gamma_{1}>0$ and $\theta >(1-\alpha_{h})/\beta_{h}$ such that


$$\begin{array}{c} g(z;u_{h},\kappa_{h})\geq \theta z,\;\forall \;z\in [0,\gamma_{1}], \end{array}$$ (23)

then **(H3)** holds by statement (iv) of Lemma 2, as the inequality in Eq (17) is satisfied. In a control-theoretic setting, the scalar $1/(c^{⊤}(I-A_{h}{)}^{-1}b_{h})$ is the reciprocal of the so-called steady state gain of the linear control system specified by the triple $\left(A_{h},b_{h},c^{⊤}\right)$. An ecological interpretation of the quantity $1/(c^{⊤}(I-A_{h}{)}^{-1}b_{h})$ is obviously context dependent, but we refer the interested reader to [77] for a nice discussion of this quantity.

Finally, as mentioned, hypothesis **(H5)** is satisfied if we choose χ(z)=1/z for z > 0.

### Main result

We are now in position to state and prove our main result of the section.

**Theorem 4.** *Consider the adaptive switching feedback control scheme* Eq (8) *with* *q* ≥ 2*, where* τ *satisfies* **(*****T)**, hypotheses* **(H1)***–* **(H5)** *hold, and the switching threshold M is chosen so that*


$$\begin{array}{c} \begin{array}{c} q_{d}^{*}=\{\text{strategies which are uniformly}\;\text{ultimately semi-globally }\eta \text{-persistent }\\ \;\text{above M and }\ell_{t}\;\text{has affine-linear growth}\}, \end{array} \end{array}$$

*is non-empty. For all x_0_ > 0 and all s_0_ > 0, the following statements apply to the solution (x,s) of* Eq (8):

(i) 𝒦(s(t)) *converges to a persistent strategy* $h_{*}$ *with persistence threshold* $\varepsilon_{h_{*}}\geq M$*, and*
(ii) ${lim inf}_{t\to \infty }\|y(t)\|\geq M$.

Before giving the proof, we provide commentary on the above theorem.

**Remark 5.** (a) In simple terms, Theorem 4 states that the adaptive switching feedback control scheme in Eq (8) selects a desirable strategy from a given, fixed collection of possible strategies — recall meaning one which results in the measured population size persisting long-term above a specified threshold, *M* — provided that such a strategy exists.

From a control-theoretic perspective, Theorem 4 is an instability result insomuch as the zero equilibrium of model Eq (1) is destabilised, which is a desirable outcome in the context of managing conserved populations. As a feedback controller, the destabilisation requires both a measured variable (the output) and qualitative descriptions (a labelling) of the strategies to be implemented. However, exact knowledge of the underlying dynamics for *x*, or the precise effects of the different strategies, are not required. Consequently, Theorem 4 has considerable robustness properties. These are discussed more fully at the end of the current section, but here we reiterate the robustness with respect to model- or parametric-uncertainty, as what is required is that the structural hypotheses **(H1)**– **(H5)** hold, and that there is at least one persistent strategy in a certain sense. We contend that these are reasonable assumptions for many common population models, including the Allen-Clark model as illustrated in Example 3.

(b) The conceptual implications of Theorem 4 are that Eq (8) may be applied to the state *x*, which is governed by uncertain positive difference equations, using a switching sequence update law of the form Eq (6). Thus, Eq (8) may be applied to a wider range of populations that are governed by uncertain dynamics than previous adaptive switching feedback control schemes and, as we will shall proceed to show, the performance can be adjusted by using different switching sequence update laws.

(c) Recall that each persistent strategy $h\in q_{p}$ has a corresponding persistence threshold $\varepsilon_{h}>0$ that acts as a lower bound for the observed population size for sufficiently large *t*. Whereas, the switching threshold *M* in the adaptive switching feedback control scheme in Eq (8) is chosen by the user to determine the minimum desirable size of the measured population, and thus also determines the magnitude of persistence threshold that is considered desirable. That is, the choice of *M* allows persistent strategies to be classed as undesirable when $M>\min_{h\in q_{d}^{*}}\varepsilon_{h}$, resulting in *q*_p_ ≠ *q*_d_. The switching sequence *s* grows under persistent strategies with a persistence threshold less than *M*, leading at some point to a future switch in strategy. Observe that a consequence of hypothesis **(H3)** is that $q_{d}^{*}$ will be nonempty if *M* > 0 is chosen sufficiently small.

(d) We comment on the adaptability of the scheme, specifically focussing on the sequences τ and *s* that are control design parameters. The rate of increase of *s* is as in Eq (7), and is crucially determined by the function χ, another design parameter. Since switching between strategies occurs as *s*(*t*) increases through τ intervals, there is consequently a coupling between these terms. In terms of the effect, intuitively, longer τ intervals or a slower increase of *s* means that strategies are applied, or tested, for longer, before switching to other strategies if needed. This might be termed a “sluggish response”. On the one hand, a sluggish response has the advantage that persistent strategies are less likely to be switched from prematurely, particularly before persistence is established, and we suspect is well suited when the dynamics in Eq (1) are themselves sluggish. However, on the other hand, a sluggish response also has the disadvantage that undesirable strategies may be persevered with for longer than necessary. This latter scenario we address with the override switching variation of the adaptive switching feedback control scheme in Eq (8) considered below. The opposite is also true; shorter τ intervals or a more rapid increase of *s*, what might be termed a “rapid response”, leads to strategies only being applied for shorter times before switching. This increases the risk of switching from persistent strategies prematurely, but reduces the risk that undesirable strategies are applied for too long. We suspect that a rapid response is more suited to dynamics that are themselves rapid. We reiterate that Theorem 4 applies in both cases, and these deliberations relate to transient performance. Overall, though, the adaptive switching feedback control scheme in Eq (8) has considerable adaptability through tuning of the terms τ and *s*, and performance is likely to be improved the more that is known about the underlying dynamics in Eq (1).

*Proof of Theorem 4.* We argue exhaustively. Set $q_{u}^{*}=\underset{―}{q}\backslash q_{d}^{*}=q_{u}\cup (q_{d}\backslash q_{d}^{*})$. That is, $q_{u}^{*}$ comprises two possibilities: (i) strategies which are not persistent at the threshold *M*, *q*_u_; or (ii) strategies which *are* persistent at threshold *M*, but do not satisfy the affine-linear growth property, $q_{d}\backslash q_{d}^{*}$. If the solution (*x*,*s*) is such that 𝒦(s(t)) converges to an element of $q_{d}\backslash q_{d}^{*}$, then there is nothing to prove. Therefore, it suffices to focus on *q*_u_.

Step 1: *s* is not bounded under fixed strategies in *q*_u_.

Since $h\in q_{u}$ gives rise to ${lim inf}_{t\to \infty }\|y(t;h)\|<M$, there exists a subsequence $(t_{k}{)}_{k}$ and δ>0 such that


$$\begin{array}{c} \|y(t_{k})\|<M-\delta \;\text{so that}\;\chi (\|y(t_{k})\|)>\chi (M-\delta )>0\;\forall \;k\in Z_{+}. \end{array}$$

Therefore, under strategy *h* the sequence *s* increases and eventually there will be a switch in strategy.

Step 2: 𝒦(s) cannot always avoid $q_{d}^{*}$.

We claim that *s* grows at fastest exponentially. Since τ grows faster than exponentially, *s* enters every τ interval (at least for sufficiently large times), and hence 𝒦(s(t)) takes every value in $\underset{―}{q}$.

In light of **(H1)** and **(H2)** (both with $\Gamma =\{x_{0}\}$ and $R>\|x_{0}\|$), for simplicity we may assume that **(H4)** holds with *m* = *k*, relabelling the constant *c* if needed. Now let $t_{0}\in Z_{+}$ denote a time that strategy $h\in q_{u}$ is (re)applied. We may assume that *t*_0_ ≥ *k*, where *k* is as in **(H4)**. Combining **(H2)** and **(H4)** yields λ>0 and γ∈(0,1) such that


$$\begin{array}{c} \|y(j+t_{0}+k)\|\geq c\|x(j+t_{0})\|\geq c\lambda \gamma^{j}\|x(t_{0})\|\;\forall \;j\in Z_{+}, \end{array}$$

and note that $\|x(t_{0})\|>0$ by **(H2)**, as $\|x(0)\|=\|x_{0}\|>0$ by hypothesis. Therefore, as χ is decreasing


$$\begin{array}{c} \chi (\|y(j+t_{0}+k)\|)\leq \chi (c\lambda \gamma^{j}\|x(t_{0})\|)\;\forall \;j\in Z_{+}. \end{array}$$

An application of hypothesis **(H5)** to the above inequality yields positive constants *a* and *b* > 1 such that


$$\begin{array}{c} \chi (\|y(j+t_{0}+k)\|)\leq \chi (c\lambda \gamma^{j}\|x(t_{0})\|)\leq ab^{j}\;\forall \;j\in Z_{+}. \end{array}$$ (24)

and which, note, are independent of *j*.

The standard telescoping sequence equality applied to switching sequence *s* reads


$$\begin{array}{c} s(t+t_{0}+k)-s(t_{0}+k)=\sum_{j=0}^{t-1}(s(j+1+t_{0}+k)-s(j+t_{0}+k))\;\forall \;t\in Z_{+}. \end{array}$$

In light of the definition of *s* in Eq (7), it follows that


$$\begin{array}{c} s(t+t_{0}+k)-s(t_{0}+k)=\sum_{j=0}^{t-1}r(\|y(j+t_{0}+k)\|\;;M)\;\forall \;t\in Z_{+}. \end{array}$$

Invoking the definition of *r* and Eq (24), we estimate the above as follows:


$$\begin{array}{c} s(t+t_{0}+k)-s(t_{0}+k)\leq \sum_{j=0}^{t-1}\chi (\|y(j+t_{0}+k)\|)\leq \sum_{j=0}^{t-1}ab^{j}\\ =a\frac{b^{t}-1}{b-1}\leq \frac{a}{b-1}b^{t}\;\forall \;t\in Z_{+}. \end{array}$$ (25)

In particular, the estimate Eq (25) entails that *s* grows at fastest exponentially, as claimed.

The conjunction of steps 1 and 2 is that, unless switching has stopped, every strategy in $\underset{―}{q}$ will be entered at some point, including strategies in $q_{d}^{*}$.

Step 3: *s* becomes bounded under a strategy in $q_{d}^{*}$.

Let $t_{1}\in Z_{+}$ denote a time that strategy $h\in q_{d}^{*}$ is (re)applied. Thus, **(H3)** applies. Combining **(H1)** and **(H2)** with $\Gamma :=\{x_{0}\}$ yields


$$\begin{array}{c} \lambda \gamma^{t_{1}}\|x_{0}\|\leq \|x(t_{1})\|\leq \Delta , \end{array}$$

for some λ,Δ>0 and γ∈(0,1). In particular, we have $x(t_{1})\in \Gamma_{\gamma }(t_{1})$ as in Eq (11) with $\delta =\lambda \|x_{0}\|>0$. Therefore, using **(H3)** and the affine-linear growth property Eq (12), there exist constants *c*,*d* > 0 such that


$$\begin{array}{c} \ell_{t_{1}}\leq c+t_{1}d. \end{array}$$

By the persistence hypothesis **(H3)** and choice of switching threshold *M*, the sequence *s* is constant after $\ell_{t_{1}}+t_{1}$ time steps, as $\|y(t+\ell )\|\geq \varepsilon_{h}\geq M$ for all $t\in Z_{+}$, by hypothesis. However, in light of Eq (25) we have that $t\mapsto s(t+\ell_{t})$ grows at fastest exponentially, whilst assumption **(T)** ensures that τ grows faster than exponentially. Hence, for sufficiently large *t*_1_, the sequence *s* becomes constant whilst $h\in q_{d}^{*}$, so is trivially bounded there, and no further switching occurs. The proof is complete. □

### Accommodating measurement noise

So far, it is assumed that the measurements taken are exact. In reality, it is likely that they will be subject to some measurement error. Here, we provide sufficient conditions under which the conclusions of Theorem 4 apply to the adaptive switching feedback control scheme Eq (8), even when the measured variable *y* is subject to (some level of) measurement noise. This result is presented as Theorem 6 below.

We model the measurement error as being proportional to the observation taken. To do this, we set


$$\begin{array}{c} y(t)=(1+\xi (t))\eta (x(t)). \end{array}$$ (26)

Here, ξ(t) is unknown and represents the measurement error at time *t*, and ξ(t)=0 corresponds to the error-free case. This measurement noise propagates into the adaptive switching feedback control scheme Eq (8) via ‖y(t)‖ and χ(‖y(t)‖).

We introduce the following assumption on ξ.

**(H6)** There exist $\xi_{\min}\in (-1,0]$ and $\xi_{\max}\geq 0$ such that $\xi (t)\in [\xi_{\min},\;\xi_{\max}]$ for all *t*.

The assumption that $\xi_{\min}>-1$ preserves the positivity of observations, whilst $\xi_{\min}\leq 0$ ensures that measurement noise does not always result in over counting. The smaller $\xi_{max}-\xi_{min}\geq 0$, the more accurate the measurements taken.

**Theorem 6.** *Consider* Eq (8) *with y given by* Eq (26) *and impose the notation and assumptions of Theorem 4, only replacing* $q_{d}^{*}$ *by*


$$\begin{array}{c} \begin{array}{c} q_{d}^{*}=\{\text{strategies}\;\text{which are uniformly ultimately semi-globally }\eta \text{-persistent }\\ \;\text{above }(1+\xi_{min})M\;\text{and }\ell_{t}\;\text{has affine-linear growth}\}, \end{array} \end{array}$$

*In this case, the conclusions of Theorem 4 hold.*

The proof is very similar to that of Theorem 4, *mutatis mutandis*, and is hence omitted. Basically, the persistence property combined with the assumptions on ξ mean that the switching threshold *M* must be less than $(1+\xi_{min})\varepsilon_{h}$ for persistence threshold $\varepsilon_{h}$ of strategy *h*, to ensure that this strategy is deemed desirable by Eq (8) when subject to measurement error. The moral of the result is that the adaptive switching feedback control scheme is robust with respect to possibly persistent, but sufficiently small, measurement errors.

**Remark 7.** *In the remainder of the work, we propose variations of the adaptive switching feedback control scheme* Eq (8), *which could also include measurement noise* Eq (26). *We present only one rigorous result for these variations, and instead explore numerical simulations and their use in examples for the others. The motivation for this choice is twofold and is: (i) to illustrate the principles at work across a range of variations, and (ii) because we expect that further rigorous results could be derived, at least under suitable assumptions, but doing so would involve repeated, but minor, variations of the arguments used in the proof of Theorem 4. Moreover, the results would be asymptotic in nature, when here transient performance is likely of more interest in practical settings.*

### Exploiting additional information in adaptive switching feedback control schemes

When undertaking a management program using the adaptive switching feedback control scheme, measurements are taken at each time step. Therefore, as the length of the program increases, there is more information available for decision making. The switching sequences considered hitherto, as well as those in [65], make use of the current measurement *y*(*t*) *only* in assessing whether *s* should increase. We begin by providing details of switching sequence update laws that use more of the available population data. We note that this is not an exhaustive list of possible adaptations to the switching sequence update law. In fact, and at least under hypotheses **(H1)**– **(H5)**, we expect any update law that results in the switching sequence increasing when in an undesirable position, but no faster than exponentially, and remaining constant when in a desirable position to be suitable. Furthermore, we provide pseudocodes that detail how to change the switching system, so that strategies can be switched out of quicker when in an undesirable position, and so that strategies that have already been rejected are avoided, respectively.

#### Moving averages.

Calculating the moving average of the observed population size and comparing this to the switching threshold can help to smooth out the effects of measurement errors or noise, as well as capture the average behaviour of oscillatory solutions. Thus, we expect a switching sequence update law that uses the moving average to be less sensitive to measurement noise and be more lenient when considering oscillatory populations. We propose averaging the measured population vectors, *y*(*t*), over the last *t*_*a*_ time steps, which we denote $a(t)\in R_{+}^{p}$, via


$$\begin{array}{c} a(t):=\left\{ \begin{array}{ccc} & \frac{1}{t+1}\sum_{i=0}^{t}y(i), & t<t_{a},\\ & \frac{1}{t_{a}}\sum_{i=0}^{t_{a}-1}y(t-i), & t\geq t_{a}, \end{array}\right.\;t\in Z_{+}. \end{array}$$ (27)

In words, for the first $t_{a}-1$ time steps, all the measured population vectors are summed and then divided by the number of time steps that have passed. Then, once *t*_*a*_ time steps have passed, the population vectors measured for the previous *t*_*a*_ time steps are summed and then divided by *t*_*a*_.

Consequently, we can set χ (see Eq (7)) to be a function of *a*(*t*). Thus, the switching sequence Eq (7) is replaced by


$$\begin{array}{c} s(t+1)=s(t)+\left\{ \begin{array}{cccc} & 0, & M\leq & \|a(t)\|,\|a(t)\|=0,\\ & \chi (\|a(t)\|), & & 0<\|a(t)\|<M, \end{array}\right.\;s(0)=s_{0},\;t\in Z_{+}. \end{array}$$ (28)

We use this type of switching sequence update law in the second and last sections of the simulation results. Furthermore, we note that all of the following adaptations to the switching system could also use ‖a(t)‖ in place of ‖y(t)‖.

A rigorous result for the moving average variation of the adaptive switching feedback control scheme in Eq (8) follows readily from Theorem 4, and is presented next as the following corollary.

**Corollary 8.** *Consider the moving average adaptive switching feedback control scheme, that is,* Eq (8) *with y(t) replaced by a(t) as given in* Eq (27)*. Under hypotheses* **(H1)***–* **(H5)***, the conclusions of Theorem 4 hold, with* $\varepsilon_{h_{*}}$ *replaced by* $\varepsilon_{h_{*}}/t_{a}$ *and y(t) replaced by a(t).*

For ease of presentation, Corollary 8 is based on a persistence threshold and the persistence hypothesis **(H3)** for the measured variable *y*, rather than for the moving average *a*. This may be conservative in practice, as for oscillatory populations the average may persist at a higher level, as is the case in the Allen-Clark model in the simulation results to come. To formulate a result based on persistence thresholds for the moving average *a* would require a version of hypothesis **(H3)** to hold for *a*.

*Proof of Corollary 8.* The proof of Theorem 4 is followed, with *y*(*t*) replaced by *a*(*t*) as given in Eq (27). Note that, by definition


$$\begin{array}{c} a(t)\geq (1/t_{a})y(t),\;\forall \;t\in Z_{+}, \end{array}$$ (29)

and so by **(H4)** for *y*, it follows that


$$\begin{array}{c} \|a(t)\|\geq (1/t_{a})\|y(t)\|\geq (c/t_{a})\|x(t-m)\|\;\forall \;t\in Z_{+},\;t\geq \max\{t_{a},k\}, \end{array}$$

that is, *a* satisfies **(H4)**.

Moreover, hypothesis **(H3)** ensures the existence of $h\in \underset{―}{q}$ which is uniformly ultimately semi-globally η-persistent with corresponding *ℓ* as in Eq (10) satisfying the affine-linear growth property. Therefore, the inequality in from Eq (29) implies (with a slight abuse of terminology) that $h\in \underset{―}{q}$ is also uniformly ultimately semi-globally *a*-persistent with the same *ℓ*. The result follows. □

#### Recent trends.

Another way in which the switching sequence update law can be adjusted so that more of the available data is used, rather than just *y*(*t*), is to compare the measured data from multiple time steps with each other. If the observed population size, ‖y(t)‖, is much smaller than the switching threshold, *M*, when a new strategy is entered, then it may take a long time before ‖y(t)‖≥M. This presents a risk that a desirable strategy may be left prematurely. To mitigate against this risk, we propose updating the switching sequence using Eq (7) in the first time step (*t* = 0); then, for all other time steps using


$$\begin{array}{c} s(t+1)=s(t)+\left\{ \begin{array}{cccc} & \chi (\|y(t)\|), & 0<\|y(t)\|<M & \;\text{and }\|y(t)\|<\|y(t-1)\|\\ & 0, & & \text{otherwise}, \end{array}\right.\;t\in N, \end{array}$$ (30)

which is constructed so that *s* only increases if ‖y(t)‖ is less than both *M* and ‖y(t−1)‖. In other words, as with the switching sequence update laws considered thus far, if the measured population size is above the switching threshold, the switching sequence will remain constant even if the measured population size has decreased over the last time step. However, unlike the switching sequence update laws considered thus far, if the measured population size has increased over the last time step, then the switching sequence will remain constant, even if the measured population size is less than the switching threshold.

We provide commentary for situations when the asymptotic dynamics differ from the transient dynamics. When there is transient growth before asymptotic decay, during the transient window ‖y(t)‖≥‖y(t−1)‖. Hence, by Eq (30), *s* would remain constant during the transient window, even if ‖y(t)‖<M. Once the transient window has ended, the asymptotic dynamics would kick in and so ‖y(t)‖<‖y(t−1)‖; thus, once ‖y(t)‖ is also less than *M*, the switching system will begin to increase. Thus, in situations such as this, the recent trends update law may take longer to switch out of a strategy corresponding to asymptotic decay than the original update law would. In other words, the recent trends update law may have a more sluggish response than the original update law.

Conversely, there could be transient decay before asymptotic growth. Thus, during the transient window ‖y(t)‖<‖y(t−1)‖. Furthermore, because we will have entered the strategy when ‖y(t)‖<M, this will result in *s* increasing over the transient window. After the transient window is over, ‖y(t)‖≥‖y(t−1)‖ and so *s* will stop increasing. If the τ interval is shorter than the transient window (which could be the case early in the run of the switching system) then there would be a switch in strategy and so we may have prematurely switched out of a desirable strategy. We note that the original update law would also switch out of the strategy in this circumstance. However, if the τ interval is longer than the transient window, then *s* would remain constant once out of the transient window when using Eq (30). Alternatively, if using the original update law, even when the transient window is over and ‖y(t)‖ is growing, there could be a switch in strategy before ‖y(t)‖≥M. Thus, in this situation, the recent trends update law can prevent a premature switch in strategy.

Finally, we comment that when the strategy corresponds to a model that has an oscillatory solution, as with the original update law, if the minimum of the oscillations is greater than *M*, then the recent trends update law will class the strategy as desirable. If instead the minimum of the oscillations is below *M*, then, as with the original update law, the recent trends update law will cause *s* to increase over a subsequence. However, the recent trends update law will only increase *s* over the subsequence where ‖y(t)‖ is less than both *M* and ‖y(t−1)‖ (rather than just when ‖y(t)‖<M) and so will take longer to switch out of the oscillatory strategy than the original update law would.

We use the recent trends switching sequence update law in the last section of the simulation results.

#### Multiple switching thresholds.

The switching sequence update laws considered so far only use two levels to assess the measured population data against the switching threshold, *M*. That is, for each time step, we are either in a desirable position or an undesirable position, and all undesirable positions increase the switching sequence by χ(‖y(t)‖). However, granularity may be added to the switching sequence update law so that the increase of *s* depends on how close ‖y(t)‖ is to *M*. This is achieved here by introducing multiple switching thresholds that are less than the original switching threshold, leading to the switching sequence update law Eq (6) being replaced by


$$\begin{array}{c} r(z;M,\beta ):=\left\{ \begin{array}{cccc} & 0, & M_{1}\leq z & ,\;z=0,\\ & \chi_{1}(z), & M_{2}\leq z & <M_{1},\\ & \;\;\vdots & & \vdots \\ & \chi_{\beta -1}(z) & M_{\beta }\leq z & <M_{\beta -1},\\ & \chi_{\beta }(z), & 0<z & <M_{\beta }. \end{array}\right. \end{array}$$

Here: β≥1 is the number of switching thresholds applied; $\chi_{1}(z)\leq \chi_{2}(z)\leq \cdots \leq \chi_{\beta }(z)$ are decreasing functions; and $M_{1}\geq M_{2}\geq \cdots \geq M_{\beta }$. Note that if $M_{1}=M_{2}=\cdots =M_{\beta }$ and $\chi_{1}=\chi_{2}=\cdots =\chi_{\beta }$, then the original switching sequence update law Eq (6) is recovered. The original switching threshold is denoted by *M*_1_ and indicates the level at which the observed population is considered to be desirable, then the additional switching thresholds are denoted by *M*_*i*_ where i∈{2,…,β}. Furthermore, each switching threshold *M*_*i*_ would correspond to a function $\chi_{i}(\|y(t)\|)$ that causes a bigger increase to the switching sequence as *i* increases (and ‖y(t)‖ decreases). In the special case that β=2, then the update Eq (7) for the switching sequence is replaced by *s*(0) = *s*_0_ and


$$s(t+1)=s(t)+\left\{ \begin{array}{lll} 0, & M_{1}\leq ∥y(t)∥, & ∥y(t)∥=0\\ \chi_{1}(∥y(t)∥), & M_{2}\leq ∥y(t)∥<M_{1}, & \\ \chi_{2}(∥y(t)∥), & 0<∥y(t)∥<M_{2}, & \end{array}\right.\;t\in {\mathbb{Z}}_{+}.$$ (31)

We envisage that switching sequence update laws with multiple switching thresholds will allow for the rate of switching to be fine-tuned resulting in strategies to be switched out of quicker when the observed population size is small, due to the higher penalties that are applied by the $\chi_{i}$ functions as *i* increases. Furthermore, we note that switching sequence update laws with multiple switching thresholds can be used alongside switching sequence update laws that use the moving average and/or the recent trends update laws. We use a switching sequence update law with two switching thresholds in the last section of the simulation results.

#### Override switching.

When the τ intervals are long, larger jumps in the value of the switching sequence will be required to cause a switch in strategy and, roughly speaking, it will take longer to do so. Evidently, applying an undesirable strategy for a long time over a “large” τ interval could result in the population size decreasing significantly before a switch occurs. To protect against this, the adaptive switching feedback control scheme may be altered so that, if the measured population size has decreased for the past *t*_o_ time steps (for some chosen, fixed *t*_o_), then a switch of strategy is enforced, independently of *s*. This is a separate issue to changing the switching sequence update law but, intuitively, is likely to decrease the time taken to determine a desirable strategy. Algorithm 1 details how to override switching. We have used this process in the last section of the simulation results.

We comment that under this regime, it is possible that an override could occur during a desirable strategy. This could happen if *t*_o_ is picked to be smaller than the transient window, which could result in ‖y(t)‖ decreasing for the first *t*_o_ time steps, resulting in an override switch. Another possibility of when the override regime could cause a switch out of strategy in $q_{d}^{*}$ is when the strategy corresponds to an oscillatory solution that has a period that is greater than 2*t*_o_, as ‖y(t)‖ would be decreasing for more than *t*_o_ consecutive time steps. If it is the case that all desirable strategies would “fail” in this way, then the adaptive switching feedback control scheme Eq (8) with override switching may not converge to a desirable strategy. Thus, for a theoretical result which guarantees a desirable strategy is identified asymptotically to be expected to hold, *t*_o_ must be picked sufficiently enough.

**Algorithm 1: Override switching**

1. Set *t* = 0, *s*(*t*)=*s*_0_ and *y*(*t*)=*y*_0_. Define $t_{o}\in Z_{+}$ to be number of time steps that *y* can decrease before overriding the switching system and forcing a switch in strategy.

2. Calculate 𝒦(s(t)) to determine which strategy is applied at time *t*.

3. Run the adaptive switching feedback control scheme to calculate *s*(*t* + 1). Measure the population to update *y*(*t* + 1).

4. If *t* ≥ *t*_o_:

• If $𝒦(s(t))=𝒦(s(t-1))=\cdots =𝒦(s(t-t_{o}+1))$ (i.e., have been in the same strategy for the last *t*_o_ time steps):

– If $\left\|y(t+1)\|<\|y(t)\|<\cdots <\|y(t-t_{o})\right\|$ (i.e., ‖y‖ is strictly decreasing over the last *t*_o_ time steps):

* Identify the largest τ(j) that is smaller than *s*(*t* + 1) (i.e., identify the lower bound of the curren*t* τ interval).

* Set s(t+1)=τ(j+1)+0.01 (i.e., force a switch by moving to the next τ interval).

* Increment *t* by 1 and return to step 2.

Else (corresponding to ‖y‖ not strictly decreasing over the last *t*_o_ time steps):

* Increment *t* by 1 and return to step 2.

Else (corresponding to having switched strategy within the last *t*_o_ time steps):

– Increment *t* by 1 and return to step 2.

Else (corresponding to *t* < *t*_o_):

• Increment *t* by 1 and return to step 2.

#### Discarding rejected strategies.

In essence, when the switching system tests different strategies, it is learning whether they are desirable or not. Hence, the adaptive switching feedback control scheme Eq (8) may be adjusted so that it discards strategies which have been previously applied and switched from (what we might term rejected strategies). Again, this is a separate issue to changing the switching sequence update law, but may also decrease the time taken to determine a desirable strategy.

Recall that 𝒦(s(t)) determines which strategy is applied during time step *t*, see Eq (5). Hence, a switch has occurred between times *t* − 1 and *t* when 𝒦(s(t))≠𝒦(s(t−1)). In the current variation, we “discard” strategy 𝒦(s(t−1)) because it has been tested and switched out of. This is achieved by increasing *s*(*t*) so that it enters the next τ interval and storing strategy 𝒦(s(t−1)) as a rejected strategy, before continuing to run the adaptive switching feedback control scheme. Observe that it is possible to discard desirable strategies. If we have discarded all strategies before the switching sequence has converged, then the adaptive switching feedback control scheme is reset so that all strategies are available again. Algorithm 2 details how to discard rejected strategies. We have used this process in the last section of the simulation results.

**Algorithm 2: Discard rejected strategies**

1. Set *t* = 0, *s*(*t*)=*s*_0_ and *y*(*t*)=*y*_0_. Initialise vector $𝐮=[\begin{array}{ccc} 1 & \cdots & q \end{array}]$ to set all strategies as available.

2. Calculate 𝒦(s(t)) to determine which strategy is applied at time *t*.

3. If 𝒦(s(t))=𝒦(s(t−1)) (i.e., have not switched strategy):

• Proceed to step 5.

Else (corresponding to 𝒦(s(t))≠𝒦(s(t−1)) – i.e., a switch of strategy):

• Set 𝒦(s(t−1)) as a discarded strategy.

• Proceed to step 4.

4. If **u** ≠ **0** (i.e., there are available strategies):

• While 𝐮(𝒦(s(t)))=0 (i.e., 𝒦(s(t)) is a discarded strategy):

– Identify the largest τ(j) that is smaller than *s*(*t*) (i.e., identify the lower bound of the curren*t* τ interval).

– Set s(t)=τ(j+1)+0.01 (i.e., move to the next τ interval).

– Increment *j* by 1.

• Proceed to step 5.

Else (corresponding to **u** = **0** — i.e., all strategies have been discarded):

• Reinitialise vector $𝐮=[\begin{array}{ccc} 1 & \cdots & q \end{array}]$ (i.e., all strategies are available again).

• Proceed to step 5.

5. Run the adaptive switching feedback controller to calculate *s*(*t* + 1). Measure the population to update *y*(*t* + 1).

6. Increment *t* by 1 and return to step 2.

### Robustness properties and practical considerations

As indicated in Remark 5, we conclude the current section by commenting further on the robustness properties and practical considerations of the adaptive switching feedback control scheme in Eq (8). The nature of our main results, Theorems 4 and 6, ensures robustness with respect to model- and parametric-uncertainty. This is because the key hypotheses **(H1)**– **(H6)** are themselves structural, and capture reasonable dynamic behaviour of the models under consideration which may be gauged likely to hold without requiring explicit knowledge of the model, captured by *F* in Eqs (1) or (8). We repeat that we contend that this is a strength of the proposed method for conservation applications, where these two sources of uncertainty are likely to be significant and management action is often required before the dynamics of the system are fully understood [68]. The other key requirement for our main results is the existence of a suitably persistent strategy in the fixed collection of available strategies. Since the effects of strategies are assumed not be known in advance, whether this is likely to be the case will be context specific. Achieving population persistence without *any* persistent strategies being available, however, seems unreasonable in general, so this requirement is arguably also mild. (We do note here, though, that so-called *dispersal induced growth* or *dispersal driven growth* may offer an approach to population persistence without the requirement of a persistent strategy, see the Discussion.)

Theorem 6 additionally ensures that the adaptive switching feedback control scheme in Eq (8) contains certain robustness with respect to measurement errors (when modelled as acting multiplicatively), and quantifies this in terms of the maximum permitted measurement error and desired persistence threshold. Note that the treatment of measurement errors models the effects of sensor degradation. Furthermore, the formulation of Theorems 4 and 6 ensures robustness with respect to measurement delays as well, that is, where the update law for *s* in Eqs (7) and (8) is replaced by


$$\begin{array}{c} s(t+1)=s(t)+r(\|y(t-\sigma )\|\;;M),\;t\in Z_{+},\;t\geq \sigma , \end{array}$$ (32)

for some delay σ∈N (and some choice as to how to define the values s(1),…,s(σ)). That these results are still valid in the presence of measurement delays is a consequence of the construction of the crucial coupling condition between measurements *y* and states *x*, hypothesis **(H4)**. Indeed, replacing *t* by t−σ, hypothesis **(H4)** reads


$$\begin{array}{c} c\|x(t-\sigma -m)\|\leq \|y(t-\sigma )\|\;\forall \;t\in Z_{+},\;t-\sigma \geq k, \end{array}$$

so that, if hypothesis **(H4)** holds, then it also holds for the adaptive switching feedback control system in Eq (8) with delayed measurement. However, and intuitively, we expect that significant measurement delays (that is, large σ) may negatively affect transient performance of Eq (8) when compared to the delay-free (σ=0) case, as the switching sequence update law Eq (32) which determines the strategy to apply is “out of sync” with the measurements taken. It is only once the τ intervals are sufficiently large, requiring transient time to have elapsed, that the strategy “intended” by the delayed measurements will be applied currently.

To implement the adaptive switching feedback control scheme in Eq (8) practically requires the ability to implement each of the *q* available strategies, and to switch between them in accordance with Eq (8). The key hypothesis in Theorems 4 and 6 of the existence of a suitable persistent strategy, captured mathematically by the sets termed $q_{d}^{*}$ in Theorems 4 and 6 being non-empty, demonstrates that the scheme is robust to potential losses of strategies (an instance of what might be termed actuator malfunctions in this setting), *provided* that $q_{d}^{*}$ remains non-empty in such circumstances. In other words, it is possible for the scheme to accommodate the loss of persistent strategies and still select a persistent strategy, provided that at least one persistent strategy remains. In a sense, this is the deliberate aim of the discarding rejected strategies variation of Eq (8) proposed above, and the ideas discussed there broadly apply in the context of actuator malfunctions as well. However, we caution that since whether strategies are persistent or not is assumed not to be known in advance, the effects of the loss of strategies are unfortunately not possible to predict, and we expect that our main results do *not* hold in the case that there are no suitably persistent strategies available. Finally in terms of implementation, the control schemes (the controllers) proposed presently are all variants of the elementary switching sequence update law Eq (7), and so the computational requirements are minimal. We note that this is a general strength of robust controllers, as compared to optimal controllers which typically require the solution of potentially complex optimisation problems, often repeatedly, to implement.

## Simulation results

Here, we provide simulation results of the adaptive switching feedback control scheme Eq (8) and its variations which we have proposed in the previous sections. First, we use a density-dependent model and show that the control scheme is robust to measurement noise and uncertainty in initial conditions. Second, we apply the control scheme to an Allen-Clark model using the moving average switching sequence update law. Next, we apply the control scheme to a two-species Pielou model with exponential term. Finally, we use the same density-dependent model to assess the performance of twenty-four different switching systems, which each use different combinations of the adaptations to the switching system proposed earlier.

### A density-dependent population with measurement noise

We present this example as if we were running the adaptive switching feedback control scheme to aid with a conservation management program for three different populations of trout cod (*Maccullochella macquariensis*). We assume that only adult fish may be observed and the initial observed population sizes for the populations in the Murray River, Ovens River and the Bendora Dam, to 2dp, are given by


$$\begin{array}{c} y_{1}(0):=9.52,\;y_{2}(0):=0.88,\;\text{and}\;y_{3}(0):=0.03, \end{array}$$ (33)

respectively. Here, units correspond to 10^3^ female fish.

When setting up the adaptive switching feedback control scheme, we assume that there are three available strategies: strategy 1 corresponds to no management intervention; strategies 2 and 3 correspond to management actions that aim to increase the number of 1-year-old fish.

We define the switching sequence update law via


$$\begin{array}{c} s(t+1)=s(t)+\left\{ \begin{array}{cccc} & 0, & 7.5\leq \|y(t){\|}_{1} & ,\;\|y(t){\|}_{1}=0,\\ & \frac{1}{\|y(t){\|}_{1}}, & 0<\|y(t){\|}_{1} & <7.5, \end{array}\right.\;s(0)=7,\;t\in Z_{+}, \end{array}$$ (34)

where the switching threshold, *M*, is 7500 female fish. Furthermore, **(H5)** is satisfied. We define the sequence τ via


$$\begin{array}{c} \tau (j+1)=0.35+(j+2)\tau (j),\;\tau (0)=0,\;j\in Z_{+}. \end{array}$$ (35)

**Fig 2:**
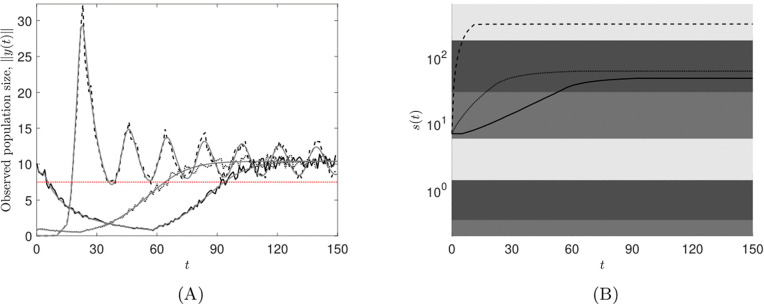
Numerical simulations of the adaptive switching feedback control scheme Eq (8) using the switching sequence update law Eq (9) for the nonlinear population model of trout cod Eq (36), with measurement noise. The first, second and third initial conditions are represented by: a solid line; a dotted line; and a dashed line, respectively. A: Trajectories of the observed population size against time *t*. Black lines represent the noisy measurements of the observed population, $(1+\varepsilon (t))\|y(t){\|}_{1}$, whilst grey lines represent the predicted total observable population, $\|y(t){\|}_{1}$, from the models. The red line is the switching threshold *M* = 7.5. B: Semilog plot to show the growth of *s* over time *t*. The medium, dark and light grey regions correspond to being in strategies 1, 2 and 3, respectively.

We plot our results in Fig 2. Each panel contains three simulations, corresponding to the initial observed population sizes recorded in Eq (33). Fig 2A displays the observed population size with measurement error, which we denote by $(1+\xi (t))\|y(t){\|}_{1}$, against time *t*. We see that for each of the initial conditions $(1+\xi (t))\|y(t){\|}_{1}$ is greater than *M* for large *t*. Fig 2B displays the growth of *s* over time and the shaded regions represent the different τ in*t*ervals. Together, Fig 2A and 2B show that, for all initial conditions, *s* becomes bounded in a strategy such that $𝒦(s(t))\to h\in q_{d}$ as t→∞ and the population persists. Indeed, we see that under strategies 2 and 3, $(1+\varepsilon (t))\|y(t){\|}_{1}>M$ as t→∞. Hence, *M* has been chosen sufficiently small such that strategies 2 and 3 are desirable. Furthermore, in Fig 2B we see that the third initial condition, *y*_3_(0) in Eq (33), results in the adaptive switching feedback control scheme having what we term a rapid response (see Remark 5d) as *s* increases more quickly than the other two initial conditions. This is due to *y*_3_(0) being much smaller than *y*_1_(0) and *y*_2_(0) as well as *s*_0_ being defined such that strategy 1 is applied first, which results in *s*(*t*) increasing more rapidly to begin with.

As mentioned, this example has been presented as if the adaptive switching feedback control scheme has been run with real-world population data. However, in actuality, the population data in this example were created using a density-dependent population projection matrix model, for each strategy h∈{1,2,3}, of the form


$$\begin{array}{c} x(t+1)=F(h,x(t))=A_{h}x(t)+b_{h}g_{h}(f_{h}^{⊤}x(t)),\;x(0)=x_{0},\;t\in Z_{+}. \end{array}$$ (36)

The model was initially developed in [78] and was adapted for use with an adaptive switching feedback control scheme in [65, Example 3.2]. The model projects the female population over an annual time step. There are seven stage classes (*n* = 7) and units correspond to 10^3^ fish. For each strategy $h\in \underset{―}{q}$: the matrix $A_{h}\in R_{+}^{n\times n}$ captures the survival and transition between stage classes; the term $b_{h}g_{h}(f_{h}^{⊤}x(t))$ models recruitment which is limited by density and hence nonlinear.

This population model fits the framework of Example 3, where conditions under which hypotheses **(H1)**– **(H5)** hold are discussed. Furthermore, it is known (from for example [65, Section 2.2] or [41, Section 3]) that the asymptotic dynamics of Eq (36) for fixed *h* are determined by the interplay of the linear data captured through the quantity


$$\begin{array}{c} p_{h}:=1/(f_{h}^{⊤}(I-A_{h}{)}^{-1}b_{h}), \end{array}$$

(which is finite and positive under mild assumptions) and the nonlinear term *g*_*h*_. Indeed, solutions are bounded if


$$\begin{array}{c} \underset{z\to \infty }{lim sup}\frac{g_{h}(z)}{z}<p_{h}, \end{array}$$

strategy $h\in q_{e}$ if


$$\begin{array}{c} \underset{z\geq 0}{\sup}\frac{g_{h}(z)}{z}<p_{h}, \end{array}$$

and $h\in q_{d}$ if


$$\begin{array}{c} \underset{z↘0}{lim inf}\frac{g_{h}(z)}{z}>p_{h}. \end{array}$$

In our simulations we have assumed that there are three available strategies for management of the species, and that they only affect the nonlinear term *g*_*h*_ in Eq (36). Our linear inputs are the same as those used in [65, Equation 3.3], which correspond to the mean life rates of the model in [78]. Furthermore, we have $\rho (A_{h})=0.8931$ and *p*_*h*_ = 0.4792 for all *h*.

**Fig 3:**
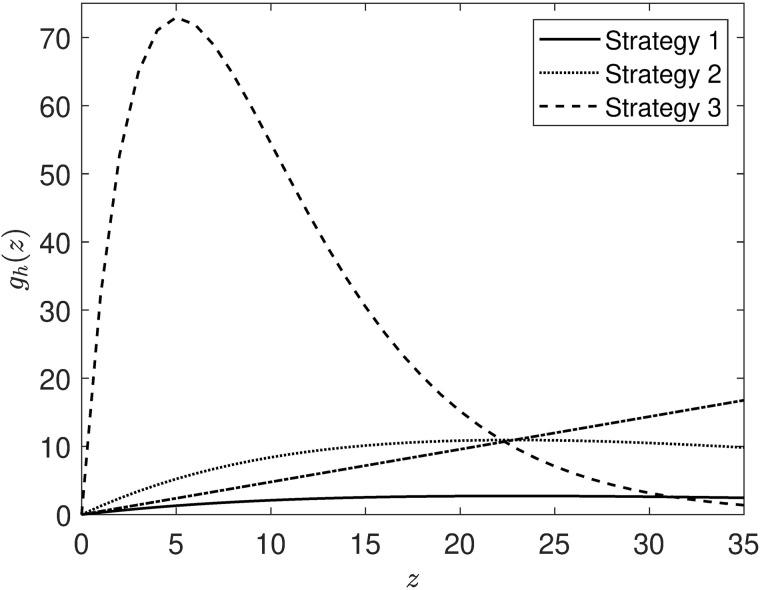
Graph of *g*_*h*_, see Eq (37) with parameters from Table 1, for h∈{1,2,3}. The dash-dot line has slope *p*_*h*_ = 0.4792 for all *h*.

The nonlinear functions we consider are of the form of a Ricker function, that is


$$\begin{array}{c} g_{h}(z)=\sigma_{h}ze^{(-1/RCC_{h})z}\;\forall \;z\geq 0,\forall \;h\in \underset{―}{q}, \end{array}$$ (37)

where $\sigma_{h}>0$ and *RCC*_*h*_ > 0 are to-be-specified positive parameters. Specifically, *RCC*_*h*_ is the carrying capacity for larval recruits. The parameter values we use for each strategy are stored in Table 1. All three strategies satisfy **(H1)** and **(H2)**. Fig 3 plots *g*_*h*_ for each h∈{1,2,3}. We see that strategy 1 is undesirable and corresponds to extinction, whilst strategies 2 and 3 are both persistent strategies. Thus, strategies 2 and 3 satisfy **(H3)**.

**Table 1:** Parameters used in the nonlinear function *g*_*h*_ in Eq (37) for the available strategies.

Strategy (*h*)	Parameters	
	$\sigma_{h}$	*RCC*_*h*_
1	0.3257	22.834
2	1.3026	22.834
3	39.078	5.0742

For our simulations we assume that the fish in each of the adult stage classes, 5–7, can be observed. Thus, η(z):=Cz with


$$\begin{array}{c} C:=(\begin{array}{ccccccc} 0 & 0 & 0 & 0 & 1 & 0 & 0\\ 0 & 0 & 0 & 0 & 0 & 1 & 0\\ 0 & 0 & 0 & 0 & 0 & 0 & 1 \end{array}). \end{array}$$ (38)

Furthermore, we have *y*(*t*) given by Eq (26), where we assume the errors ξ(t) are uniformly distributed on the interval [−0.1, 0.1]. Hence, arguing analogously as in Example 3, it can be shown that **(H4)** and **(H6)** are satisfied. Thus, the adaptive switching feedback control scheme is given by Eq (8), with *x*(*t* + 1) given by Eq (36) and *s*(*t* + 1) given by Eq (34).

The initial conditions in Eq (33) were generated using random perturbations of the unique non-zero equilibrium of Eq (36) associated with strategy 2, and denoted x^*^, meaning


$$\begin{array}{c} x^{*}:=(I-A_{2}{)}^{-1}b_{2}z^{*}\;\text{where}\;z^{*}>0\;\text{is the unique solution of}\;g_{2}(z^{*})=p_{2}z^{*}. \end{array}$$

Initial conditions 1, 2 and 3 correspond to initial population sizes of breeding females: 5,000–10,000; 500–2,500; and, 25–50, respectively. These ranges correspond to the population sizes of the Murray River, Ovens River and the Bendora Dam or Seven Creeks, respectively, as estimated in [79]. The initial population vectors used in our numerical simulations are,


$$\begin{array}{c} x_{1}(0):=(\begin{array}{c} 7.7601\\ 3.2057\\ 1.4681\\ 0.9816\\ 1.1582\\ 0.8896\\ 7.4739 \end{array}),\;x_{2}(0):=(\begin{array}{c} 0.7519\\ 0.3413\\ 0.1658\\ 0.2467\\ 0.1122\\ 0.2308\\ 0.5395 \end{array}),\;x_{3}(0):=(\begin{array}{c} 0.0274\\ 0.0154\\ 0.0105\\ 0.0055\\ 0.0037\\ 0.0034\\ 0.0234 \end{array}), \end{array}$$ (39)

where *x*_*j*_(0) represents *x*(0) for initial condition *j*.

In this example, *M* has been chosen sufficiently small such that both the persistent strategies are desirable, that is *q*_p_ = *q*_d_. Furthermore, both persistent strategies have affine-linear growth, so $q_{d}^{*}=\{2,3\}$. Therefore, the hypotheses of Theorem 6 are satisfied. The grey lines in Fig 2A show the noise-free total observable population, $\|Cx(t){\|}_{1}$, predicted when using population models of the form Eq (36).

#### Increasing the magnitude of noise.

Here, we use the same trout cod model Eq (36), and the same three strategies (see Table 1) whilst varying the magnitude of measurement noise and the switching threshold, *M*. We run the adaptive switching feedback control scheme using Eq (9) to update the switching sequence *s* and, again set *s*_0_: = 7. We test 21 different magnitudes of noise from 0 to 1 with increments of 0.05, that is,


$$\begin{array}{c} \xi (t)\in [-\xi_{\text{max}},\xi_{\text{max}}]\;\text{where}\;\xi_{\text{max}}=0.05k,\;0\leq k\leq 20. \end{array}$$

We also test 19 different values for *M*. We see in Matlab that


$$\begin{array}{c} y_{*}:=\underset{t\to \infty }{lim inf}F(3,x(t))\approx 9.5. \end{array}$$

In other words, the oscillatory solution *x* of strategy 3 has a minimum of approximately 9.5 thousand adult female fish. We choose to test values of *M* from $0.05y_{*}$ up to $0.95y_{*}$, incrementing by $0.05y_{*}$ each time, that is,


$$\begin{array}{c} M=0.05ky_{*},\;1\leq k\leq 19. \end{array}$$

For each magnitude of noise and switching threshold combination, we perform 300 simulations corresponding to different initial conditions. There are 100 different initial conditions generated for each range of initial population sizes of breeding females, which are: 5,000–10,000; 500–2,500; and, 25–50, as before.

**Fig 4:**
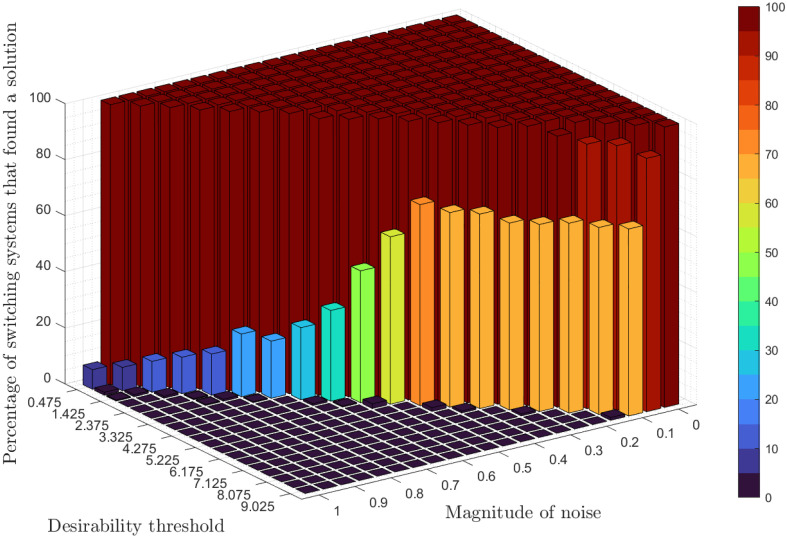
Percentage of the initial conditions for which the switching system identifies a persistent strategy when changing both the magnitude of noise, $\xi_{max}$, and the switching threshold, *M.*

We run all switching systems for 10,000 time steps and compute the number which have constant *s* for the last 100 time steps, which we use as a proxy for the number of switching systems where *s* has converged (that is, a persistent strategy has been identified). We plot the results in Fig 4.

We see that the percentage of switching systems that identify a persistent strategy decreases as the noise magnitude increases. Similarly, the percentage of switching systems that identify a persistent strategy decreases as the switching threshold increases. As predicted by Theorem 6, we see that the adaptive switching feedback control scheme Eq (8) can tolerate a certain amount of measurement noise. Moreover, as the switching threshold decreases, a larger magnitude of measurement noise can be tolerated.

#### Uncertainty in initial conditions.

Here, we demonstrate that the adaptive switching feedback control scheme Eq (8) is robust with respect to uncertainty in initial conditions. In all simulations, we use the trout cod model as in Eq (36).

First, we demonstrate that our results are robust with respect to the choice of τ, provided that **(T)** is satisfied. We use a (pseudo-)random number generator to generate 1000 uniformly distributed constants $\tau_{i}(1)\in (0,8)$ where i∈{1,2,…,1000}. Thus, instead of Eq (35), each sequence $\tau_{i}$ is defined via


$$\begin{array}{c} \tau_{i}(j+1)=\tau_{i}(1)+(j+2)\tau (j),\;\tau_{i}(0)=0,\;j\in Z_{+},\;\forall \;i\in \{1,2,\ldots ,1000\}. \end{array}$$ (40)

In words, we randomly generate the size of the first τ interval and consequently the size of the later τ intervals also vary. Setting the initial population vector to be each *x*_*j*_(0) in Eq (39) in turn, we run the adaptive switching feedback control scheme with the original switching sequence update law, that is Eq (9), and each $\tau_{i}$. For all simulations, we set *s*_0_: = 7 and *M*: = 7.5.

**Fig 5:**
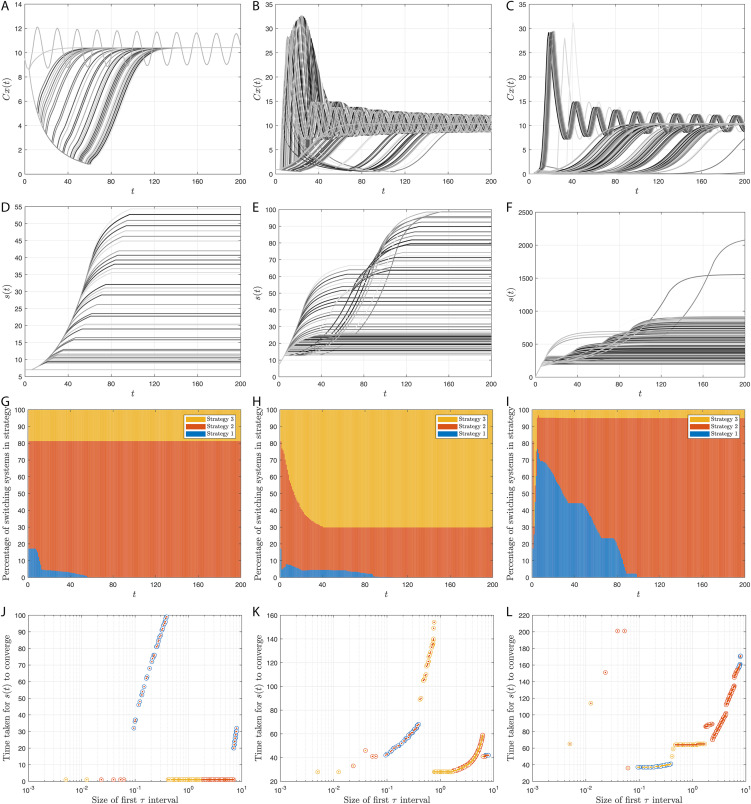
Numerical simulations of the adaptive switching feedback control scheme Eq (8) using 1000 different τ sequences generated using Eq (40). The switching sequence is updated via Eq (34) and the nonlinear population model of is given by Eq (36). The first, second and third column of subfigures plot the results for initial population vectors *x*_1_(0), *x*_2_(0) and *x*_3_(0), respectively. A–C: Trajectories of the observed population size, ‖Cx(t)‖, against time *t*. Grey scale used to distinguish between curves. D–F: Growth of *s* over time *t*. Grey scale used to distinguish between curves. G–I: Percentage of simulations in each strategy at time *t*. J–L: Time taken for *s* to become bounded by length of first τ interval, $\tau_{i}(1)$ for i∈{1,2,…,1000}. Circles, ∘, and points, ·, correspond to the strategy used in time *t* = 0 and *t* = 200, respectively.

Our results are plotted in Fig 5. We see that *s* becomes bounded in a persistent strategy with ‖Cx(t)‖>M as t→∞ for all switching systems, see Fig 5A–F. In Fig 5G–I, for initial population vectors *x*_1_(0), *x*_2_(0) and *x*_3_(0), we see that 81.3%, 29.8% and 95% of switching systems become bounded in strategy 2, respectively; with the other switching systems becoming bounded in strategy 3 (18.7%, 71.2% and 5% for initial populations *x*_1_(0), *x*_2_(0) and *x*_3_(0), respectively). Fig 5J–L show that the size of the first τ interval, $\tau_{i}(1)$, determines which strategy each switching system starts in as well as which strategy *s* becomes bounded in and how long it takes to do so. When the initial population vector is *x*_1_(0), we have that $\|Cx_{j}(0)\|\geq M$. Consequently, if the size of $\tau_{i}(1)$ corresponds to the strategy applied during the first time step, which we denote by *h*_*i*_(0), being strategy 2 or 3, then no switching will occur (see Fig 5J). If, instead, the size of $\tau_{i}(1)$ corresponds to *h*_*i*_(0) = 1 and $\|Cx_{j}(0)\|\geq M$, then the time taken for *s* to become bounded seems to increase linearly as the size of $\tau_{i}(0)$ increases. For initial population vectors *x*_2_(0) and *x*_3_(0), we know that $\|Cx_{j}(0)\|<M$ and when *h*_*i*_(0) = 1, the time taken for *s* to converge still seems to increase linearly as the size of $\tau_{i}(1)$ increases (see Fig 5K and L). However, when $h_{i}(0)\in \{2,3\}$, the relationship between the size of $\tau_{i}(1)$ and the time taken for *s* to converge is not so clear. The size of $\tau_{i}(1)$ dictates not only which strategy $𝒦(s_{0})$ corresponds to and the size of subsequent τ intervals, but also how far *s*_0_ is away from the end of the initial τ interval. Thus, the value of $\tau_{i}(0)$ influences how many switches are needed before *s* converges and how long it takes to do so.

Next, we demonstrate that our results are robust with respect to the choice of *s*_0_. We use a (pseudo-)random number generator to generate 1000 uniformly distributed constants $s_{i}(0)\in (0,14)$ and define the switching sequence update law via Eq (9). We run the adaptive switching feedback control scheme with each *s*_*i*_(0) for each of the initial population vectors in Eq (39). For all simulations, we set *M*: = 7.5, and the τ sequence is given by Eq (35).

**Fig 6:**
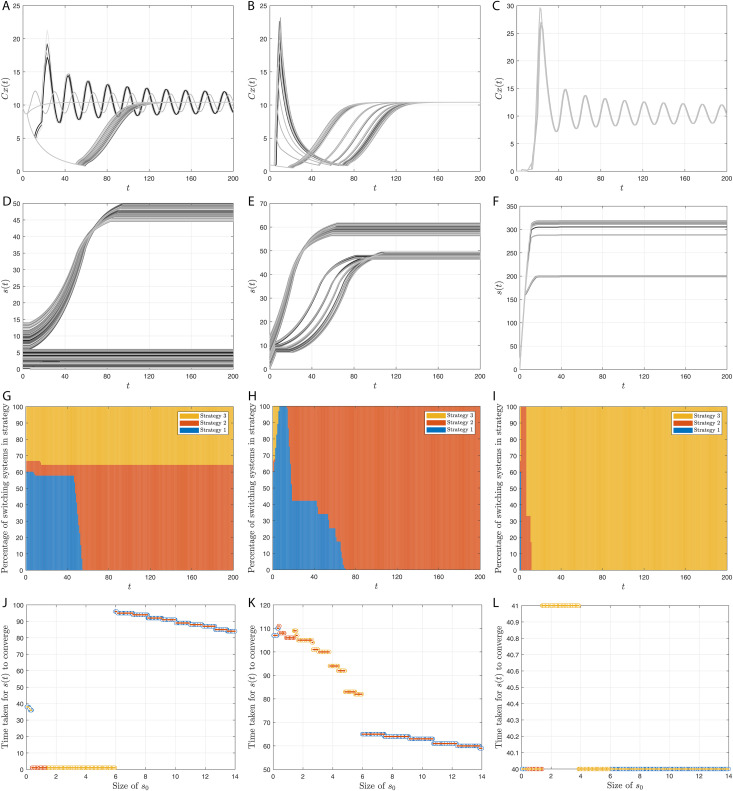
Numerical simulations of the adaptive switching feedback control scheme Eq (8) using 1000 different switching system initial conditions, *s*_*i*_(0) for i∈{1,2,…,1000}. The τ sequence is given by Eq (35), the nonlinear population model of is given by Eq (36), and the switching sequence is updated via Eq (9). The first, second and third column of subfigures plot the results for initial population vectors *x*_1_(0), *x*_2_(0) and *x*_3_(0), respectively. A–C: Trajectories of the observed population size, ‖Cx(t)‖, against time *t*. Grey scale used to distinguish between curves. D–F: Growth of *s* over time *t*. Grey scale used to distinguish between curves. G–I: Percentage of simulations in each strategy at time *t*. J–L: Time taken for *s* to become bounded by length of first τ interval, $\tau_{i}(1)$ for i∈{1,2,…,1000}. Circles, ∘, and points, ·, correspond to the strategy used in time *t* = 0 and *t* = 200, respectively.

Our results are plotted in Fig 6. We see from Figures 6A–F that *s* becomes bounded in a persistent strategy with ‖Cx(t)‖>M as t→∞ for all *s*_*i*_(0). In Fig 6G–I, we see that, when the initial population is given by *x*_1_(0), 64.4% of switching systems become bounded in strategy 2, with the other 35.6% becoming bounded in strategy 3. When the initial population is given by *x*_2_(0), all the switching systems become bounded in strategy 2 (see Fig 6H); whereas when the initial population is given by *x*_3_(0), all the switching systems become bounded in strategy 3 (see Fig 6I). Fig 6J–L show that the time taken for *s* to become bounded depends on the size of *s*_*i*_(0). The size of *s*_*i*_(0) dictates which τ interval, and consequently which strategy, the switching system starts in, see Table 2. As before, for initial population vector *x*_1_(0), when $h_{i}(0)\in \{2,3\}$ no further switching occurs and *h*_*i*_(*t*) = *h*_*i*_(0) for all $t\in Z_{+}$. If instead *h*_*i*_(0) = 1, then the time taken for *s* to become bounded decreases as *s*_*i*_(0) increases due to switching out of strategy 1 quicker, in other words the switching system has a more rapid response when *s*_*i*_(0) is further along the τ interval. For initial population vector *x*_2_(0), the time taken for *s* to become bounded tends to decrease as *s*_*i*_(0) increases; whereas, for initial population vector *x*_3_(0), the time taken for *s* to become bounded only varies by one time step for all values of *s*_*i*_(0).

**Table 2:** Initial strategy *h*_*i*_(0) is determined by *s*_*i*_(0) and the τ intervals.

	Relationship between *s*_*i*_(0) and the τ intervals			
	$0\leq s_{i}(0)\leq 0.35$	$0.35<s_{i}(0)\leq 1.4$	$1.4<s_{i}(0)\leq 5.95$	5.95 < *s*_*i*_(0) ≤ 30.1
**Initial strategy (*h*_*i*_(0))**	1	2	3	1

Finally, we illustrate that our results are robust with respect to the initial population size and structure. We use a (pseudo-)random number generator to generate 1000 vectors ${𝐩}_{i}\in R_{+}^{n}$ where the size of each entry **p**_*i*_(*m*), is uniformly distributed on the interval (0,*M*/2) for all m∈{1,2,…,n}. Then, we generate 1000 random perturbations of each of the initial population vectors, *x*_*j*_(0), in Eq (39) via


$$\begin{array}{c} x_{j}^{i}(0):=x_{j}(0)\circ {𝐩}_{i}\;\forall \;j\in \{1,2,3\},\;\forall \;i\in \{1,2,\ldots ,1000\}. \end{array}$$

For all simulations, the τ sequence is given by Eq (35), the switching sequence update law is given by Eq (9) and we set *s*_0_: = 7, *M*: = 7.5.

**Fig 7:**
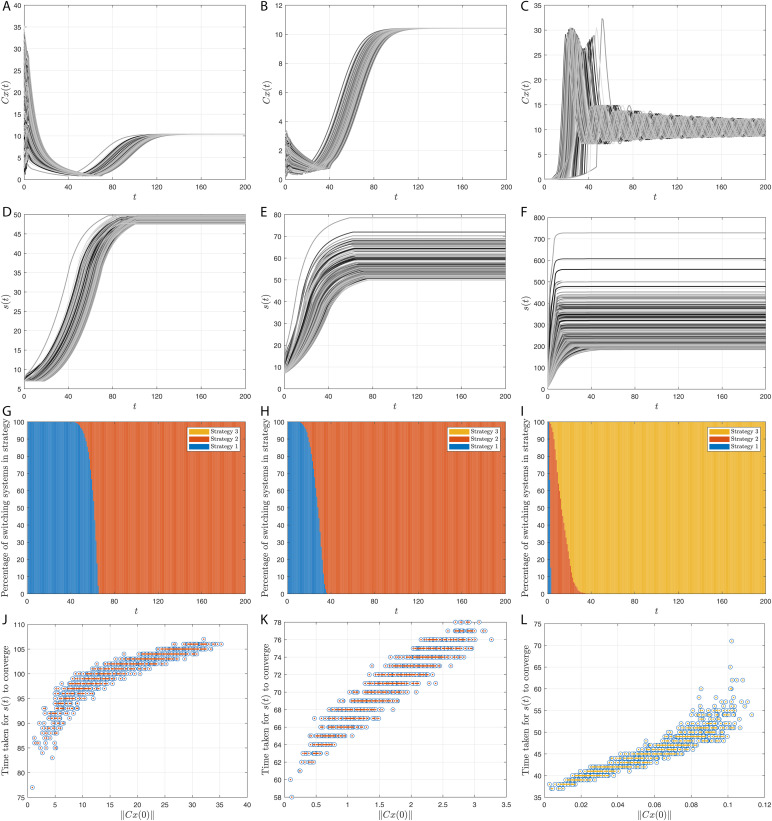
Numerical simulations of the adaptive switching feedback control scheme Eq (8) using 1000 different perturbations of each initial population vector in Eq (39). The τ sequence is given by Eq (35), the nonlinear population model of is given by Eq (36), and the switching sequence is updated via Eq (9). The first, second and third column of subfigures plot the results for initial population vectors *x*_1_(0), *x*_2_(0) and *x*_3_(0), respectively. A–C: Trajectories of the observed population size, ‖Cx(t)‖, against time *t*. Grey scale used to distinguish between curves. D–F: Growth of *s* over time *t*. Grey scale used to distinguish between curves. G–I: Percentage of simulations in each strategy at time *t*. J–L: Time taken for *s* to become bounded by length of first τ interval, $\tau_{i}(1)$ for i∈{1,2,…,1000}. Circles, ∘, and points, ·, correspond to the strategy used in time *t* = 0 and *t* = 200, respectively.

Our results are plotted in Fig 7. We see that *s* becomes bounded in a persistent strategy with $\|Cx_{j}^{i}(t)\|>M$ as t→∞ for all $x_{j}^{i}(0)$ where i∈{1,2,…,1000} and j∈{1,2,3}, see Fig 7A–F. In Fig 7G and H, we see that when the initial population vector is a perturbation of *x*_1_(0) or *x*_2_(0), *s* becomes bounded in strategy 2. Whereas, Fig 7I shows that when the initial population vector is a perturbation of *x*_3_(0), *s* becomes bounded in strategy 3. Fig 7J–L show that the time taken for *s* to become bounded increases as $\left\|Cx_{j}^{i}(0)\right\|$ increases, which increases proportionately as the size of the adult population in the initial population vector $x_{j}^{i}(0)$ increases. This is because for all simulations, the switching system starts in strategy 1 (the undesirable strategy) and so large initial adult population sizes have a more sluggish response and take longer to switch out of strategy 1.

### An Allen-Clark model

We resume our consideration of the Allen-Clark model from Example 3, now in the context of the adaptive switching feedback control scheme in Eq (8). Recall that in Example 3 we showed that the Allen-Clark model in Eq (19) satisfies **(H1)** and **(H2)** hold. Hypothesis **(H3)** holds if Eq (23) is satisfied and, furthermore, with measured variable *y* as in Eq (21), hypothesis **(H4)** holds as well.

For the present example, we assume that there are three available strategies (*q* = 3), each given by a model of the form Eq (18) with assumed two-year time delay, leading to


$$\begin{array}{c} z(t+1)=\alpha_{h}z(t)+\beta_{h}z(t-2)e^{-\kappa_{h}u_{h}z(t-2)},\;z(-j)=z_{-j},\;j=0,1,2,\;t\in Z_{+}. \end{array}$$ (41)

Here, for each strategy $h\in \underset{―}{q}$, we have: $\alpha_{h}=e^{-Z_{h}}\in (0,1)$, where *Z*_*h*_ is the overall instantaneous mortality rate; $\beta_{h}>0$ is the maximum per capita reproduction rate (at low population abundance); $\kappa_{h}$ is the density-dependent mortality near equilibrium abundance parameter; and, *u*_*h*_ is the fixed forcing term corresponding to environmental variation or harvesting. Thus, the functions $g(\cdot \;;u_{h},\kappa_{h}):R_{+}\to R_{+}$ are given by


$$\begin{array}{c} g(w;u_{h},\kappa_{h})=we^{-\kappa_{h}u_{h}w}\;\forall \;w\geq 0. \end{array}$$ (42)

**Fig 8:**
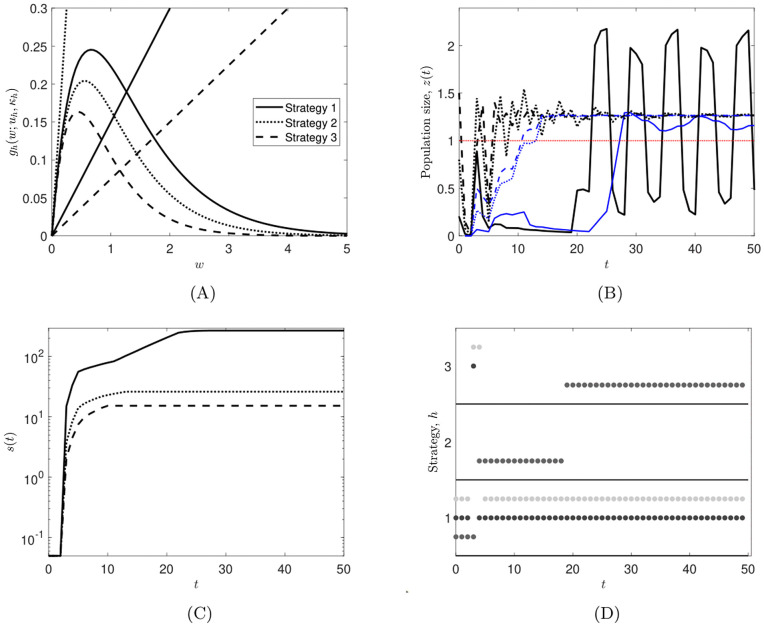
Numerical simulations of the adaptive switching feedback control scheme Eq (8) using the moving average switching sequence update law Eq (28) for the Allen-Clark model Eq (41). A: Graph of $g(\cdot \;;u_{h},\kappa_{h})$, see Eq (42), for h∈{1,2,3} using the parameter values stored in Table 3, and straight lines with gradient $p_{h}=(1-\alpha_{h})/\beta_{h}$. B: Trajectories of the population size *z*(*t*) at time *t*. Black lines plot the measured population size at time *t*. Blue lines plot the moving average calculated at time *t*. The first, second and third initial conditions are represented by: a solid line; a dotted line; and a dashed line, respectively. The red line is the switching threshold *M* = 1. C: Growth of the switching sequence *s* over time *t*. The first, second and third initial conditions are represented by: a solid line; a dotted line; and a dashed line, respectively. D: The strategy (*h*) applied at time *t*. The first, second and third initial conditions are represented by: medium, dark and light grey, respectively.

The parameters used for each model are given in Table 3. Strategy 1 is the same as the unforced Allen-Clark model described in [41, Example 6.1]. Strategies 2 and 3 have a fixed forcing term, which captures environmental variation or harvesting, at each time step. We plot the nonlinear function $g_{h}(w;u_{h},\kappa_{h})$ for each strategy in Fig 8A. We see that strategies 1 and 3 satisfy Eq (23), consequently **(H3)** is satisfied and these strategies correspond to persistence, whereas strategy 2 corresponds to extinction.

**Table 3:** Parameters used in the Allen-Clark model, in Eq (41) for the available strategies, h∈{1,2,3}.

Strategy (*h*)	Parameters			
	$\alpha_{h}$	$\beta_{h}$	$\kappa_{h}$	*u*_*h*_
1	0.1	6	1.5	1
2	0.4	0.5	2	0.9
3	0.1	12	1.5	1.5

We simulate the adaptive switching feedback control scheme with a moving average update law of the form Eq (28) for the switching sequence. We average over 5 time steps, thus *a*(*t*) is given by Eq (27) with $t_{a}:=5$. We set *s*_0_: = 0.05 and the switching threshold *M*: = 1. In our simulations the τ sequence is given by Eq (35). Thus, the adaptive switching feedback control scheme is given by Eq (8), with *z*(*t* + 1) given by Eq (41) and *s*(*t* + 1) given by Eq (28). Combined, the hypotheses of Corollary 8 are satisfied.

We perform three simulations, each corresponding to different initial conditions stored in Table 4. Fig 8B and C plot the population size, *z*(*t*), and the switching sequence, *s*(*t*), against time *t*, respectively. Fig 8D shows which strategy is applied at time *t*; that is, 𝒦(s(t)) is plotted against time *t*. We see that the switching sequences become constant, and that 𝒦(s(t)) converges to a desirable strategy. Specifically, we see that the first initial condition becomes bounded in strategy 3 whilst the other initial conditions become bounded in strategy 1. We see that strategy 1 corresponds to a stable equilibrium and persists at a level greater than the switching threshold, *M*. Strategy 3 corresponds to a oscillatory solution which spans above and below *M*; this would be deemed an undesirable strategy if we were using the original switching sequence update law used in [65]. However, the moving average of the population size remains above *M* (see the blue lines in Fig 8B), and so strategy 1 is a desirable strategy. Hence, using the moving average switching sequence update law, the switching threshold does not have to be set below the minimum of the oscillations to have the oscillatory strategy deemed to be desirable. However, if *M*, was increased such that it was still below the convergent solution, but the moving average of the oscillatory solution was not always greater than *M*, then *s*(*t*) would grow as t→∞ during the periods when the moving average was below *M*. Hence, strategy 1 would no longer be considered a desirable strategy.

**Table 4:** Initial conditions used for the numerical simulations of the Allen-Clark model Eq (41) plotted in Fig 8.

Initial condition	*z*(0)	*z*(−1)	*z*(−2)
1	0.2	0	0
2	0.8	0	0
3	1.5	0	0

### A Pielou model with exponential term

We apply the adaptive switching feedback control scheme Eq (8) to a so-called Pielou difference system with exponential term from [80], which itself is inspired by Pielou models [81]. We use population models that model two coexisting populations of interest and are of the form


$$\begin{array}{c} \begin{array}{cc} x_{1}(t+1) & =F_{1}(h,x_{1}(t),x_{2}(t))=\frac{a_{h}x_{2}(t)}{c_{h}+x_{2}(t)}e^{-x_{1}(t)}\\ \;\text{and}\;x_{2}(t+1) & =F_{2}(h,x_{1}(t),x_{2}(t))=\frac{b_{h}x_{1}(t)}{d_{h}+x_{1}(t)}e^{-x_{2}(t)} \end{array}\} \end{array}$$ (43)

which we see is of the form Eq (1) by writing


$$\begin{array}{c} x(t+1)=F(h,x(t))\;\text{with}\;F\left(h,(\begin{array}{c} x_{1}\\ x_{2} \end{array})\right)=(\begin{array}{c} F_{1}(h,x_{1},x_{2})\\ F_{2}(h,x_{1},x_{2}) \end{array}). \end{array}$$

Here: *x*_1_(*t*) and *x*_2_(*t*) denote the population size of populations 1 and 2 at time *t*, respectively; and *a*_*h*_, *b*_*h*_, *c*_*h*_ and *d*_*h*_ are positive constants for each $h\in \underset{―}{q}$. We assume that there are two available strategies (*q* = 2), which are taken from [80, Example 4.1 and Example 4.2], and record the parameter values in Table 5. We assume that both populations are measured exactly, that is, *y*(*t*): = *x*(*t*).

**Table 5:** Parameters used in the Pielou model, in Eq (43) for the available strategies, h∈{1,2}.

Strategy (*h*)	Parameters			
	*a*_*h*_	*b*_*h*_	*c*_*h*_	*d*_*h*_
1	0.8	0.9	0.6	0.5
2	0.6	0.5	0.8	0.9

Miao & Zhang [80, Theorem 2.1 (i)] provide sufficient conditions that guarantee the boundedness of solutions of Eq (43) for fixed *h*. Similarly, [80, Theorem 3.3] yields that strategy 1 results in persistence, whilst strategy 2 results in the extinction of both populations. Hence, strategies 1 and 2 are desirable and undesirable, respectively.

For completeness, in Section 2 in S1 Appendix we verify that hypotheses **(H1)**– **(H4)** hold for the Pielou model in Eq (43).

The switching sequence update law requires some adjustments so that both populations are considered. Specifically, we adapt the original switching sequence update law so that we are in a desirable position when both populations are above the switching threshold, *M*. More precisely, Eq (9) is replaced with *s*(0) = *s*_0_ and


$$\begin{array}{c} s(t+1)=s(t)+\left\{ \begin{array}{cccc} & 0, & (\min_{i}|x_{i}(t)|=0)\cup (\min_{i} & |x_{i}(t)|\geq M),\\ & \frac{1}{\min_{i}|x_{i}(t)|}, & 0<\min_{i} & |x_{i}(t)|<M, \end{array}\right.\;\forall \;t\in Z_{+}. \end{array}$$ (44)

Thus, the adaptive switching feedback control scheme is given by Eq (8) with

- ‖y(t)‖ replaced by $\min_{i}|x_{i}(t)|$, and χ(z):=1/z;
- *x*(*t* + 1) given by Eq (43), and;
- *s*(*t* + 1) given by Eq (44).

Hypothesis **(H5)** is satisfied, and hence so are all the hypotheses of Theorem 4.

**Fig 9:**
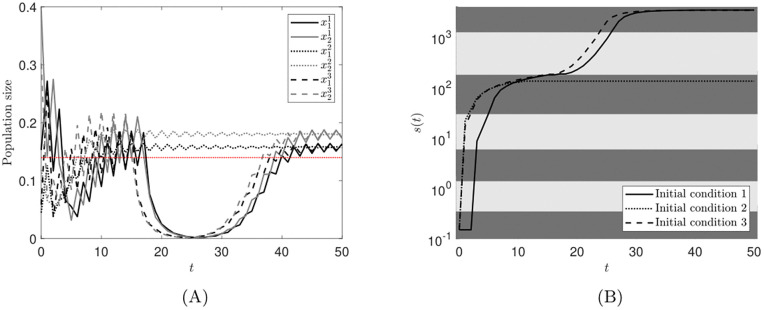
Numerical simulations of the adaptive switching feedback control scheme Eq (8) using the switching sequence update law Eq (44) for the two population Pielou model with exponential term Eq (43). The first, second and third initial conditions are represented by: a solid line; a dotted line; and a dashed line, respectively. A: Trajectories of the two populations’ size, $\|x_{1}(t){\|}_{1}$ and $\|x_{2}(t){\|}_{1}$ at time *t*. The initial condition is denoted by an *i* superscript, such that $\|x_{1}^{i}(t){\|}_{1}$ denotes the size of population 1, with initial condition *i*. The red line is the switching threshold *M* = 0.14. B: Semilog plot to show the growth of *s* over time *t*. The dark and light grey shaded regions correspond to being in strategy 1 and 2, respectively.

We perform three simulations, each corresponding to different initial conditions given in Table 6. All simulations use *s*_0_: = 0.15, *M*: = 0.14 and the sequence τ is defined as in Eq (35). Fig 9A plots the two populations’ size, |*x*_1_(*t*)| and |*x*_2_(*t*)|, against time *t*. Fig 9B plots the switching sequence, *s*(*t*), against time *t* with shaded regions representing the different τ intervals. We see that, for all initial conditions, the switching sequences become constant, and that *s* becomes bounded whilst in strategy 1, the desirable strategy, where |*x*_1_(*t*)| and |*x*_2_(*t*)| both persist above the switching threshold. Hence, the adaptive switching feedback control scheme has selected a strategy where both populations persist.

**Table 6:** Initial conditions used for the numerical simulations plotted in Fig 9.

Initial condition (*i*)	$x_{1}^{i}(0)$	$x_{2}^{i}(0)$
1	0.1530	0.03942
2	0.0449	0.1188
3	0.0586	0.3024

### Comparing different constructions of the switching system

Here, we compare and contrast the different switching system constructions, characterised by using different combinations of the adaptations to the switching system we proposed earlier. Table 7 details the construction of the 24 different switching systems.

**Table 7:** Details of how the 24 distinct switching systems used in the final section of the simulation results are defined. Switching system update laws used include: the original update law; the moving averages (MA) update law; and the recent trends (RT) update law. Additional changes used include: multiple switching thresholds (MST); the override (OR) algorithm; and the discard rejected strategies (DRS).

Switching system	Switching sequence update law			Additional changes		
	Eq (9)	Eq (28)	Eq (30)	Eq (31)	Algorithm 1	Algorithm 2
	original	MA	RT	MST	OR	DRS
1	✓					
2	✓			✓		
3	✓				✓	
4	✓					✓
5	✓			✓	✓	
6	✓			✓		✓
7	✓				✓	✓
8	✓			✓	✓	✓
9		✓				
10		✓		✓		
11		✓			✓	
12		✓				✓
13		✓		✓	✓	
14		✓		✓		✓
15		✓			✓	✓
16		✓		✓	✓	✓
17			✓			
18			✓	✓		
19			✓		✓	
20			✓			✓
21			✓	✓	✓	
22			✓	✓		✓
23			✓		✓	✓
24			✓	✓	✓	✓

We use the same trout cod model with three strategies that appeared in the first example in the simulation results, but assume that there is no measurement noise. The observed population is given by y(t)=η(x(t))=Cx(t), where *C* is as in Eq (38). Thus, **(H1)**– **(H4)** hold and strategies 2 and 3 satisfy **(H3)**.

Switching systems 1–8 use the original switching sequence update law Eq (9); switching systems 9–16 use a moving average (MA) switching sequence update law, see Eq (28); and switching systems 17–24 use the recent trends (RT) switching sequence update law Eq (30). For the MA update law, Eq (28) is evaluated with *t*_*a*_ = 5; in other words the population size is averaged over the previous 5 time steps. All update laws used satisfy **(H5)** and we set *M* = 7.5 for all switching systems. Thus, the hypotheses of Theorem 4 are satisfied.

Some of the switching systems are also subject to additional changes, such as: the switching sequence update law containing multiple switching thresholds (MST); overriding (OR) the switching system, see Algorithm 1; and/or discarding rejected strategies (DRS), see Algorithm 2. Specifically, switching systems 2, 5, 6, 8, 10, 13, 14, 16, 18, 21, 22, 24 are subject to multiple switching thresholds; switching systems 3, 5, 7, 8, 11, 13, 15, 16, 19, 21, 23, 24 use Algorithm 1 to override the switching system; and switching systems 4, 6, 7, 8, 12, 14, 15, 16, 20, 22, 23, 24 use Algorithm 2 to discard rejected strategies. Again, we refer the reader to Table 7 for a visual overview of the different switching systems used.

The switching systems that have multiple switching thresholds include one additional switching threshold; hence the switching sequence update law is of the form Eq (31). In particular, the switching sequence update law increases *s* by $\chi_{1}(y(t)):=\chi (y(t))$ when $\|y(t){\|}_{1}$ is below the switching threshold, *M*_1_: = *M*: = 7.5, unless $\|y(t){\|}_{1}$ is also below the additional switching threshold, *M*_2_: = *M*/2 = 3.75, in which case *s* increases by $\chi_{2}(y(t)):=2\chi (y(t))$. Overall, the switching sequence update law is given by


$$s(t+1)=s(t)+\left\{ \begin{array}{ll} 0, & ∥y(t)∥\geq M,\;{∥y(t)∥}_{1}=0\\ \frac{1}{{∥y(t)∥}_{1}}, & \frac{M}{2}\leq {∥y(t)∥}_{1}<M,\\ \frac{2}{{∥y(t)∥}_{1}}, & 0<{∥y(t)∥}_{1}<\frac{M}{2}, \end{array}\right.\;s_{\left(0\right)}=s_{0},\;t\in {\mathbb{Z}}_{+}.$$

Hence, the switching sequence increases by twice as much when $\|y(t){\|}_{1}$ is less than half of the original switching threshold, *M*, than it does when greater than *M*/2 but less than *M*. The multiple switching thresholds are applied similarly when using the MA update law Eq (28), except that the switching thresholds *M*_*i*_ are compared to $\|a(t){\|}_{1}$ rather than $\|y(t){\|}_{1}$, and $\chi_{i}$ is a function of *a*(*t*) instead of *y*(*t*), for all i∈{1,2}. When using the RT update law, with *s*(0)=*s*_0_ and *t* ≥ 1, the multiple switching thresholds are evaluated via


$$s(t+1)=s(t)+\left\{ \begin{array}{ll} 0, & {∥y(t)∥}_{1}\geq {∥y(t-1)∥}_{1},\;∥y(t)∥\geq M,\;{∥y(t)∥}_{1}=0,\\ \frac{1}{{∥y(t)∥}_{1}}, & {∥y(t)∥}_{1}<{∥y(t-1)∥}_{1}\;\text{and }\frac{M}{2}\leq {∥y(t)∥}_{1}<M,\\ \frac{2}{{∥y(t)∥}_{1}}, & {∥y(t)∥}_{1}<{∥y(t-1)∥}_{1}\;\text{and }0<{∥y(t)∥}_{1}<\frac{M}{2}\text{.} \end{array}\right.$$

In other words, the switching sequence increases by twice as much when $\|y(t){\|}_{1}$ is less than both switching thresholds and $\|y(t-1){\|}_{1}$ than it does when $\|y(t){\|}_{1}$ is less than $\|y(t-1){\|}_{1}$ and between the switching thresholds. Furthermore, the switching sequence remains constant when $\|y(t){\|}_{1}$ is greater than or equal to either $\|y(t-1){\|}_{1}$ or *M*.

We set $t_{o}:=10$ for all the switching systems that use Algorithm 1. Hence, if the population size has been decreasing, whilst in the same strategy, for 10 time steps then Algorithm 1 forces a switch in strategy.

**Fig 10:**
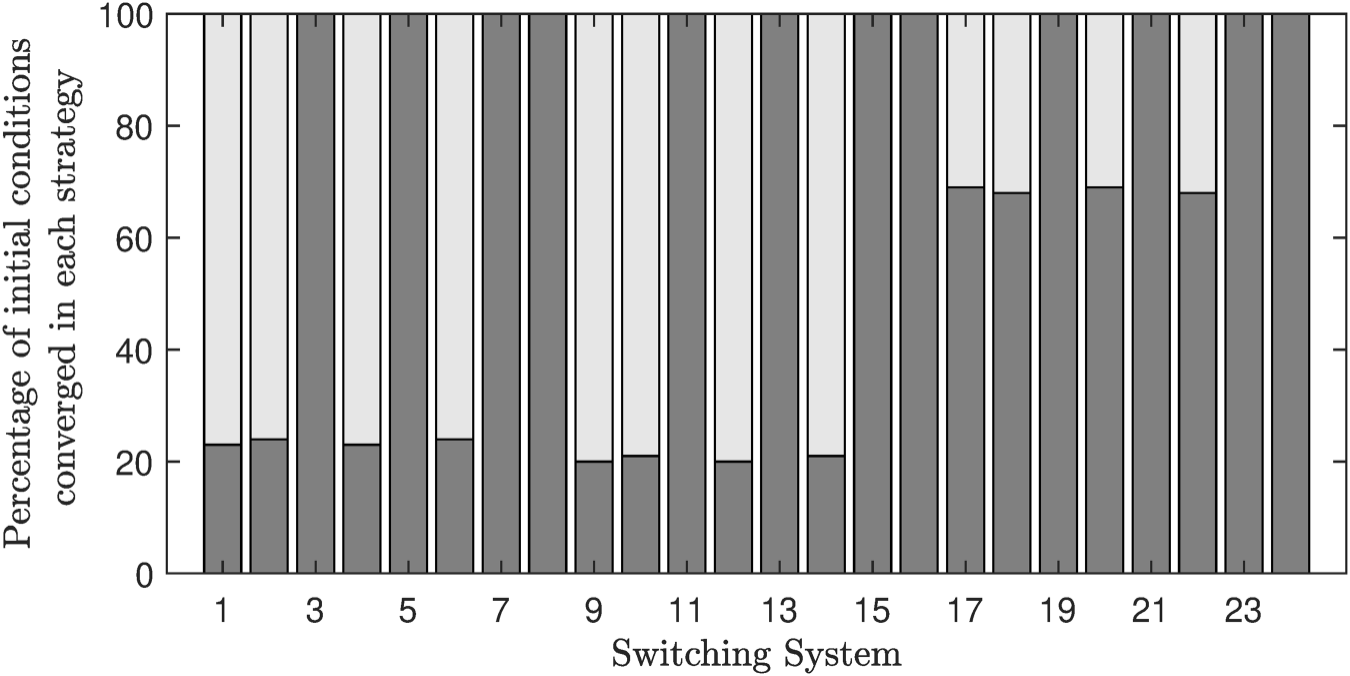
Percentage of the initial condition runs that converge to each strategy. Dark and light grey indicates converged in strategy 2 and 3, respectively. No runs converge in strategy 1, the undesirable strategy.

**Fig 11:**
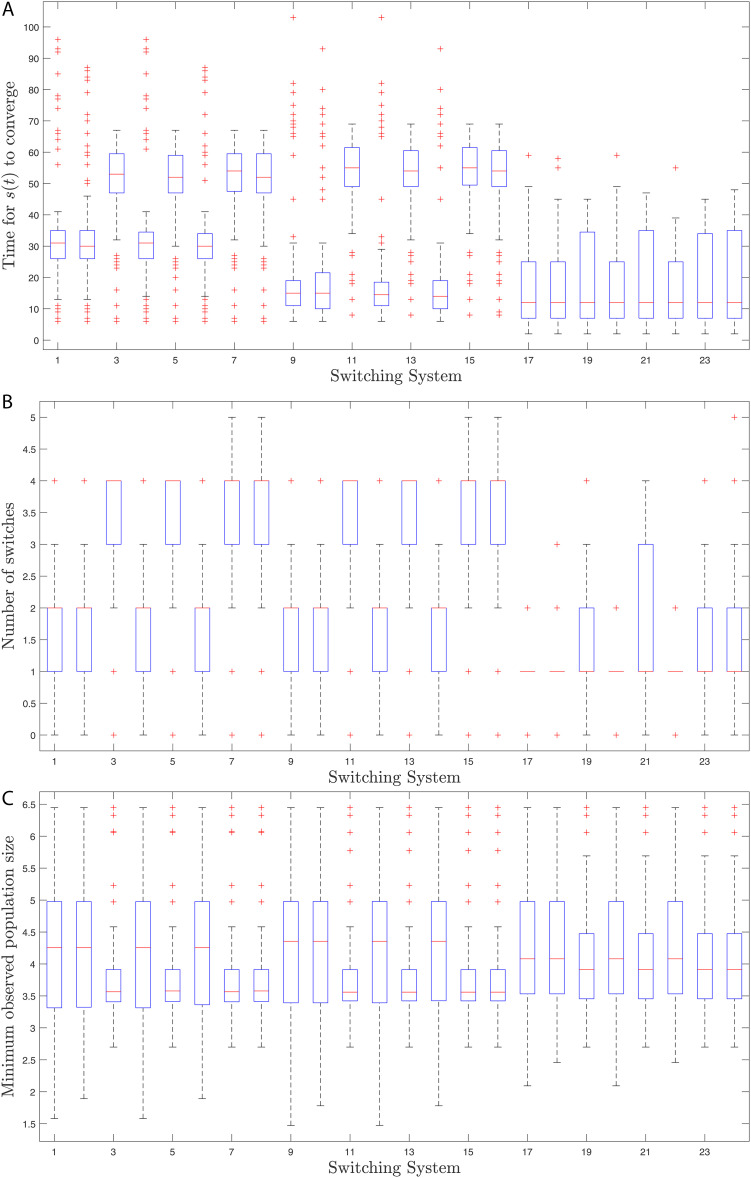
Box plots for the switching systems detailed in Table 7. For each switching system, 100 sets of initial conditions for *x*_0_, *s*_0_, and τ(1) are tested. A: Time taken for *s* to converge by switching system type. B: Minimum observed population size reached before *s* converged, by switching system type. C: Number of times the switching system switched strategy before *s* converged, by switching system type.

We assess the performance of the 24 switching systems recorded in Table 7 using 100 different sets of initial conditions by comparing: the time taken for *s* to converge; the minimum observed population size reached before *s* converges; and the number of switches before *s* converges. The initial conditions *x*_0_, *s*_0_ and τ(1) are generated using a random number generator in Matlab and each switching system is run using the same 100 sets of initial conditions, which are recorded in an online repository (see the Data Availability statement). Our results are plotted in Figs 10 and 11. We provide further details of the summary statistics in Section 3 in S1 Appendix.

In Fig 10, we see that, for all switching systems, all initial conditions converge in a desirable strategy, with 68.75% converging in strategy 2 and 31.25% converging in strategy 3. Hence, all 24 switching systems have selected a persistent strategy for all 100 sets of initial conditions. Switching systems that use the original, MA and RT update laws converge in strategy 2 on 61.75%, 60.25% and 84.25% of runs, respectively. Switching systems that use MST converge in strategy 2 on 68.83% of runs, switching systems that use the OR algorithm all converge in strategy 2, and switching systems that use the DRS algorithm converge in strategy 2 on 68.75% of runs. Whereas switching systems that do not apply any additional changes converge in strategy 2 on 37.33% of runs.

**Time to convergence:** In Fig 11A, we see that, for the 100 initial conditions tested, the switching systems that use the RT update law Eq (30), on average, take the least time for *s* to converge; median time to convergence is 12 for all switching systems that use update law Eq (30). This is because the RT update law ensures that *s* does not increase when ‖y(t)‖ is growing. Therefore, if the population size is smaller than *M* but growing we do not have to wait to be in a τ interval large enough to achieve ‖y(t)‖>M for *s* to stop increasing. Instead, *s* can become bounded in smaller τ intervals and so, here, the inclusion of the RT update law improves the response time of the switching system. Whereas the switching systems that use original and MA update laws have a median time to convergence of 37 and 28, respectively. In other words, switching systems that use the RT update law, on average, have a more rapid response than switching systems that do not.

Switching systems 9 (MA and no additional changes) and 12 (MA and DRS) are the only switching systems that correspond to an initial condition with a maximum time to convergence that is greater than 100 time steps. Hence, these switching system set ups have the most sluggish response.

Although switching systems that use the OR algorithm have the highest median time for *s* to converge (48 compared to 28 for MST and 27 for DRS), the maximum time taken for *s* to converge is much smaller when using the OR algorithm (69 compared to 93 for MST and 103 for DRS). Hence, the response time of switching systems that use the OR algorithm is more consistent than those that use MST or the DRS algorithm.

**Minimum population size:** In Fig 11B, we see that the switching systems that include the OR algorithm have the largest minimum observed population size at 2.7 thousand female fish. This is likely because, no matter the size of the current τ interval, the population size will only be allowed to decrease for a maximum of 10 consecutive time steps before a switch is forced. Due to there only being two strategies in this example, this means that switches are most likely to be forced from the undesirable strategy into the desirable strategy resulting in less time spent in an undesirable strategy and so the population size does not decrease so much.

Switching systems 9 and 12, which both use the MA update law, have the smallest minimum observed population size at 1.47 thousand female fish. However, switching systems 9 and 12, along with switching systems 10 and 14, which also use the MA update law, have the highest median minimum observed population size at 4.35 thousand female fish. Despite this, switching systems that use the MA update law have a smaller median minimum observed population size (3.38 thousand female fish) than switching systems that use the original update law (3.73 thousand female fish) and the RT update law (3.93 thousand female fish). This is likely because when ‖y(t)‖<M and the population size is monotonically decreasing, the MA update law delays the time taken for *s* to be increased compared to the original and RT update laws. For example, if we have


$$\begin{array}{c} \|y(t)\|<\|y(t-1)\|<\cdots <\|y(t-4)\|<M \end{array}$$

then, at time *t*, the original and RT update laws would increase *s* by $\frac{1}{\left\|y(t)\right\|}$, whereas the MA update law would increase *s* by


$$\begin{array}{c} \frac{1}{\|a(t){\|}_{1}}:=\frac{5}{\sum_{i=0}^{4}\|y(t-i)\|}\leq \frac{1}{\left\|y(t)\right\|}. \end{array}$$

Hence, the observed population size has to decrease more for the MA update law to increase *s* by the same amount as the original or RT update laws.

**Number of switches:** In Fig 11C, we see that the highest number of switches taken for *s* to converge is five and the smallest is zero. The only switching systems that take up to five switches to select a persistent strategy are 7, 8, 15, 16 and 24, of which 7 and 15 use the OR and the DRS algorithms and switching systems 8, 16 and 24 also use both algorithms along with MST. This is because by design the OR algorithm forces switches, and so is likely to switch more than when not using the OR algorithm. Furthermore, when the OR algorithm is paired with the DRS algorithm, the switching system not only switches more but also discards strategies that have been switched out of. Thus, if both the desirable strategies have been discarded, then the switching system will be forced to enter the undesirable strategy and so will have to switch again, at which point all strategies become available again.

Switching systems that used the RT update law require on average 1 switch to select a persistent strategy. Switching systems 1, 2, 4, 6, 9, 10, 12 and 14 require on average 2 switches. These are the switching systems that use the original update law, or the MA update law combined with either: no additional changes; MST; the DRS algorithm; or MST and the DRS algorithm. Switching systems 3, 5, 7, 8, 11, 13, 15 and 16 require on average 3 switches. These are the switching systems that use the original update law, or the MA update law combined with either: the OR algorithm; MST and the OR algorithm; the OR and DRS algorithms; or MST and both algorithms. We see that the median number of switches required for switching systems that use the OR algorithm is 3, whereas switching systems that use MST and/or the DRS algorithm require a median of 2 switches to identify a persistent strategy. Hence, this example suggests that using the OR algorithm will, on average, require more switches to identify a persistent strategy than when not using it.

Interestingly, the initial conditions that switched five times before selecting a persistent strategy do not correspond to the maximum time taken for *s* to converge. The initial conditions that switched five times when switching system 7 was applied took 56 and 59 time steps to converge, whereas the maximum time taken to converge when switching system 7 was applied was 67 time steps. Similarly, the initial conditions that switched five times before converging when switching system 15 was applied took 58 and 60 time steps to converge, whereas the maximum time taken to converge when switching system 15 was applied took 69 time steps. The initial conditions that switched five times before converging when switching systems 8 and 16 were applied both took 60 time steps to converge. The maximum time taken to become bounded when switching systems 8 and 16 were applied were 67 and 69 time steps, respectively. However, the initial condition that switched five times before converging when switching system 24 was applied took 48 times steps, which is also the maximum amount of time that switching system 24 took to converge.

## Discussion

A novel suite of adaptive switching feedback control schemes, of the form or based upon Eq (8), has been proposed and studied. The purpose of these control schemes is to identify desirable strategies from a discrete set of options, and where the effects of each strategy is not known in advance. The current work is in the spirit of robust control as it is designed to achieve its control objectives in the presence of substantial uncertainty. The adaptive switching control schemes are feedback systems as a measured variable is required, and this variable is used to update the switching sequence that ultimately determines which strategy is applied. Desirable strategies correspond to those which give rise to population persistence, and the motivating application is to conservation although the theory applies to other dynamic, managed resources. Here a strategy corresponds to what might be termed a management action or policy intervention.

The present work substantially generalises and improves our earlier contribution [65], in two directions. On the one hand, here the assumed underlying population models, captured by F(h,·) in Eq (1) are not specified exactly, and rather are required to satisfy rather mild and natural dynamic (namely boundedness and exponential decay) and persistence properties, captured in the hypotheses **(H1)**– **(H3)**. As with any feedback scheme using a measurement (or output) variable which may not be the whole state, some coupling between the measurements and states is required, here captured in **(H4)**. We have shown that the adaptive switching feedback control scheme can be applied to Allen-Clark and Pielou models that include delay recruitment and so do not fit the specific form of the Lur’e systems considered in [65]. We note that the positive difference equations used in this paper do not comprise an exhaustive list of models that satisfy the hypotheses of our main results. On the other hand, several innovations of the switching sequence update law have been proposed. These adaptations include: using more information available to the feedback control scheme when deciding whether to switch strategy; inserting an override to the switching sequence update law; discarding strategies which are perceived to be undesirable; using multiple levels of switching thresholds to include granularity; and using averaged outputs to suppress the effects of oscillatory populations. Very roughly speaking, these adaptations are designed to influence the transient performance of the adaptive switching feedback control scheme. Overall, our results show that the switching systems under consideration are widely applicable and can be tailored to the requirements of the user.

Our first main theoretical result is Theorem 4 which, roughly speaking, states that under suitable assumptions the adaptive switching feedback control scheme Eq (8) identifies a desirable strategy, assuming one is present, ensuring asymptotic population persistence. The second main result is Theorem 6 which shows that Eq (8) is quantitively robust to some level of measurement noise — a likely situation in applied scenarios. The robustness properties of the adaptive switching feedback control scheme have been discussed, and illustrated across our simulation results. For instance, in the first section of the simulation results, we demonstrate the robustness of Eq (8) to the choice of the design parameters *s*_0_ and τ(1), as well as the initial population vector *x*(0). We see that for all τ(1), *s*_0_ and *x*(0), *s* becomes bounded in a desirable strategy although the time taken and number of switches required to do so varies. We view robust control to be an advantageous tool in this setting, as often conservation actions need to be taken quickly to reduce the risk of population declines, and so there is little time to improve the knowledge of the system [68].

The remainder of the paper presents further adaptations to the switching sequence update law summarised above, with numerical and computational results. As we note in Remark 7, we expect that rigorous theoretical results for the later innovations could be established, but is not the present focus in part owing to the likely similarity with Theorem 4 and the expected asymptotic nature of these results. In the examples presented, we see that the adaptations to the adaptive switching feedback control scheme can improve the transient performance of the switching system. In the second section of the simulation results, we see that the moving averages update law Eq (28) allows for oscillatory solutions to be more easily assigned as desirable strategies. In the last section of the simulation results, we see that on average, the recent trends update law Eq (30) identifies a persistent strategy much quicker than the original update law Eq (9); median time to convergence is 12 time steps when using Eq (30) compared to 37 time steps when using Eq (9).

Furthermore, in the third section of the simulation results, we provide a switching sequence update law that can be used when there are multiple populations of interest. This setup requires the desirability conditions to be specified for all populations separately. Provided that the model assumptions are satisfied by all population models, then a solution will be obtained where all populations persist. We envisage that this type of switching sequence update law could be beneficial in multi-species conservation programs and mixed fisheries.

There are a few key principles underpinning our results. The first is that the hypotheses on the models under consideration and the construction of the switching sequence *s* yields that *s* can only grow at fastest exponentially. However, the sequence τ is constructed to grow faster than exponentially. This deliberate mismatch in rates of change is crucial. Indeed, it has the upshot that all strategies must be “visited” by the model, including the desirable strategies, and the assumed persistence dynamics of desirable strategies (captured as the *affine-linear growth* property) ensures that “a persistence threshold can be reached” before a τ interval is left and the strategy switches again (these properties all holding at least for large enough times). Once a persistence threshold is reached under a desirable strategy, switching ceases. We comment that the underlying technical assumptions are both mild and reasonable for a number of population models, as evidenced across our examples. With these key principles, we have greatly expanded the situations (models and switching sequences) which may be treated, as compared to [65]. Moreover, the present results are applicable to positive dynamical systems and positive control systems more widely, that is, in contexts outside of conservation. Indeed, the adaptive switching feedback control scheme could be beneficial in the wide range of fields that use positive dynamical systems, including: biology, physics, medicine, demography, economics, computer science, sociology as well as civil and electrical engineering [30,82].

### 4.1. Future research

Here, we briefly discuss four strands of future research which naturally follow from the current study.

(I) The first strand is to characterise, or quantify with respect to certain parameters, the transient behaviour of the adaptive switching feedback control scheme and its variations. Transient effects are a key consideration in conservation (and theoretical ecology more broadly [83]), yet arguably cannot be quantified with a single metric, and so this is a challenging goal which likely requires more information on the to-be-controlled system. Such additional assumptions, of course, are somewhat at odds with the current framework of assuming that considerable uncertainty is present, but may be applicable in certain contexts. Indeed, in these cases such additional investigations could provide valuable insight that informs how to set up the control scheme(s) the most effectively. Somewhat relatedly, one limitation of the current work is that the theoretical results only ensure persistence asymptotically, whilst transient behaviour is likely to be of considerable importance to practitioners. We propose that in future work, switching could instead depend on the transient dynamics. It is known from the dispersal driven growth (also known as dispersal induced growth) literature, that dispersal between coupled populations, that would become extinct when isolated, can cause population persistence [84,85]. Thus, we envisage that a new adaptive switching feedback control scheme could couple strategies, corresponding to asymptotic extinction but transient growth, in such a way that, moving between strategies results in population persistence. This would require that the strategies (or at least some of them) exhibit transient growth. Using this new control scheme, the assumption that there is at least one persistent (and desirable) strategy could be relaxed, as the switching system could prescribe how to switch between strategies, that correspond to asymptotic extinction, to achieve long-term population persistence through switching.
(II) We note that in many scenarios where mathematical systems and control theory have been used in population management the work has focussed on either optimal control techniques (e.g., [86–90]) or highly-robust control algorithms (e.g., [48,76,91,92]), but where performance or optimality is not addressed. Although there are a few studies where robust optimal control techniques have been applied in population management (e.g., [93–96]), it is a long-standing aim to design control-theoretic tools for ecological management and decision making which strike a middle ground between robustness and optimality. Thus, future research could seek to use ideas from optimal control theory to improve the performance of the adaptive switching feedback control scheme by, for instance, minimising the time taken to identify a persistent strategy. Alternatively, the objective of an optimal adaptive switching feedback control scheme could be to minimise the long term cost of maintaining a persistent population.
(III) Another avenue which could be explored is to augment the adaptive switching feedback control schemes presented here with tools from systems identification to (attempt to) learn the underlying dynamics. In more detail, whilst the adaptive switching feedback control scheme is used to determine a persistent strategy, another control scheme could be used in parallel to approximate the underlying function that governs the dynamics of the state, *x*, under each proposed strategy. Over time, the learned dynamics may be used to enhance the performance of the switching feedback control scheme. The system identification branch of the control scheme could use an adaptive approximation-based control design using neural networks or fuzzy systems. In their book [97], Qi, Tao and Jiang provide a systematic framework for identification and adaptive control of fuzzy systems which may provide a useful starting point.
(IV) The adaptive switching feedback control schemes considered are based upon deterministic populations models (possibly with uncertain or stochastic measurement error). Therefore, future research could focus on applying the adaptive switching feedback control scheme to stochastic models to better portray how the control scheme is applicable to real-world systems and explore the extent to which it is robust when there is uncertainty within the dynamics of the state. Finally, experimental validation of the theoretical and computational results would advance the potential practical deployment of the adaptive switching feedback control schemes considered presently.

### 4.2. Conclusion

In closing, we have presented a robust adaptive switching feedback control scheme for uncertain positive systems subject to discrete control strategies, as well as several variations which can be tailored to the requirements of the user. The motivating application is to conservation of managed populations, but the theoretical results apply more generally to positive dynamical systems. Furthermore, we have shown that the control scheme has considerable robustness properties, including with respect to model- and parametric-uncertainty, potential loss of control strategies, measurement delays, and measurement errors. We believe that with these generalisations, the adaptive switching feedback control scheme can be a valuable robust control tool when seeking to ensure the persistence of managed resources.

## Supporting information

S1 Appendix Technical details and supporting information for the simulation results. (PDF)
