# Supplementary material for: Learning under uncertainty—Conservation of populations and persistence of dynamic resources through adaptive switching feedback controllers
Source: PLoS One. 2026 Jun 1;21(6):e0349236. doi: 10.1371/journal.pone.0349236 (PMC13225672; doi:10.1371/journal.pone.0349236)
Supplement: S1 Appendix — (PDF) [file pone.0349236.s001.pdf]

S1 Appendix

Phoebe Smith and Chris Guiver

22nd April 2026

We provide technical details not given in the main text, as well extra material for the final section of our simulation results.

The software code to produce the examples can be found at `https://github.com/psjs22/Switching2`.

1 Proof of Lemma 2

We note that here, we use Eq(x) to refer to equations from the main text, where x is the equation number as it appears in the main text. Whereas, when referring to equations that appear here, but not in the main text, we use Eq(1.y), where y refers to the equation number within this section of S1 Appendix.

Proof of Lemma 2. We record here that for strictly positive $w \in \mathbb{R}^n_+$, the function

$$\mathbb{R}^n \to \mathbb{R}_+, \quad z \mapsto w^\top |z| = \sum_{j=1}^{n} w_j |z_j|, \quad \forall\, z \in \mathbb{R}^n\,,$$

is a norm on $\mathbb{R}^n$, with values equal to $w^\top z$ when $z \in \mathbb{R}^n_+$. By norm equivalence, it follows that $w^\top|\cdot|$ is equivalent to $\|\cdot\|$. We shall use this property repeatedly in the arguments to come.

For all statements, let bounded $\Gamma \in \mathbb{R}^n_+$ (bounded as well by some fixed $R > 0$) and $v : \mathbb{Z}_+ \to \underline{q}$ be given. Let x denote the solution of Eq(13), with $x(0) \in \Gamma$.

(i) Since $\rho(X_1) < 1$, we may choose strictly positive $w \in \mathbb{R}^n_+$ and $\mu \in (0,1)$ such that $w^\top X_1 \le \mu w^\top$, and define $W(t) := w^\top x(t)$ for all $t \in \mathbb{Z}_+$. Applying $w^\top$ to both sides of Eq(13), we estimate that

$$\begin{aligned} W(t+1) = w^\top x(t+1) = w^\top F(v(t), x(t)) &\le w^\top \big(X_1 x(t) + X_2\big) \\ &\le \mu w^\top x(t) + w^\top X_2 = \mu W(t) + c_0, \quad \forall\, t \in \mathbb{Z}_+\,, \end{aligned}$$

where c_0 is a nonnegative constant. An elementary induction argument gives that W satisfies the variation of parameters inequality

$$W(t) \le \mu^t W(0) + \sum_{j=0}^{t-1} \mu^{t-1-j} c_0 = \mu^t W(0) + \frac{c_0(1-\mu^t)}{1-\mu}, \quad \forall\, t \in \mathbb{Z}_+\,. \tag{1.1}$$

Since Γ is bounded, the right-hand side of the above inequality is bounded, independently of $x(0) \in \Gamma$ and $v : \mathbb{Z}_+ \to \underline{q}$. We conclude that **(H1)** holds, as $W(t)$ is equivalent to the norm of $\|x(t)\|$.

(ii) & (iii) By **(H1)**, there exists $d > 0$ such that $\|x\|$ is bounded from above by d. Now let irreducible $A_d \in \mathbb{R}^{n\times n}_+$ be as in Eq(15). Let $w \in \mathbb{R}^n_+$ denote a strictly positive left-eigenvector of A_d corresponding to $\theta := \rho(A_d) \in (0,1)$, the existence of which is ensured under irreducibility of A_d by the Perron-Frobenius Theorem (see, for instance, [1, Theorem 1.4, p.27]). Then Eq(15) yields

$$w^\top F(h,z) \ge w^\top A_d z = \theta w^\top z \quad \forall\, z \in \mathbb{R}^n_+,\ \|z\| \le d, \quad \forall\, h \in \underline{q}\,. \tag{1.2}$$

Defining again $W(t) = w^\top x(t)$ for all $t \in \mathbb{Z}_+$, it now follows from Eq(1.2) that

$$W(t+1) = w^\top F(v(t), x(t)) \ge \theta W(t), \quad \forall\, t \in \mathbb{Z}_+\,,$$

since $\|x(t)\| \le d$ for all $t \in \mathbb{Z}_+$. Hence, W admits the lower bound

$$W(t_1 + t_2) \ge \theta^{t_1} W(t_2), \quad \forall\, t_1, t_2 \in \mathbb{Z}_+\,,$$

from which **(H2)** follows by norm equivalence of $W(t)$ and $\|x(t)\|$.

To establish **(H4)**, we invoke that η is non-decreasing and Eq(15) to estimate

$$\begin{aligned}\|y(t)\| = \|\eta(x(t))\| &= \big\|\eta\big(F(v(t-1), x(t-1))\big)\big\| \\ &\geq \|\eta(A_d x(t-1))\| \quad \forall\, t \in \mathbb{Z}_+, t \geq 1\,.\end{aligned}$$

Repeating the above estimate $\nu - 1$ times yields

$$\|y(t)\| \geq \|\eta(A_d^\nu x(t-\nu))\| \geq c\|x(t-\nu)\| \quad \forall\, t \in \mathbb{Z}_+,\ t \geq \nu\,, \tag{1.3}$$

where we have invoked the inequality in Eq(16). (The above inequality follows immediately from Eq(16) if $\nu = 0$.) We conclude that **(H4)** holds with $k = m = \nu$.

(iv) It is convenient to first prove that Eq(1) is uniformly ultimately semi-globally id-persistent, that is, where $y = x$, and such that ℓ admits the affine-linear growth property. For which purpose, since the inequality in Eq(14) is assumed to hold, the argument in the proof of statement (i) above applies, and particularly the upper bound in Eq(1.1) is valid. Therefore, we see that $\|x(t)\| \leq \gamma_0$ for all $t \in \mathbb{Z}_+$, for some $\gamma_0 > 0$ as Γ is bounded by $R > 0$. Observe that γ_0 is independent of Γ. Let irreducible $A_{\gamma_0} \in \mathbb{R}_+^{n\times n}$ be as in Eq(15).

Let $\gamma_1 > 0$ and irreducible $A_{\gamma_1} \in \mathbb{R}_+^{n\times n}$ be as in Eq(17), and write $\rho_1 := \rho(A_{\gamma_1}) > 1$. Further, let $w_1 \in \mathbb{R}_+^n$ denote a strictly positive left-eigenvector of A_{γ_1} corresponding to ρ_1. The conjunction of $w_1^\top \gg 0$ and irreducibility of A_{γ_0} (which, in particular, implies that it has no zero columns) entails that $w_1^\top A_{\gamma_0} \gg 0$.

We proceed to bound the solution x of Eq(13) from below. Define $W(t) := w_1^\top x(t)$ for all $t \in \mathbb{Z}_+$. We argue exhaustively.

Case I $\|x(t)\| \leq \gamma_1$. In this case, the inequality in Eq(17) yields

$$W(t+1) = w_1^\top x(t+1) = w_1^\top F(h, x(t)) \geq w_1^\top A_{\gamma_1} x(t) = \rho_1 w_1^\top x(t) = \rho_1 W(t)\,.$$

Case II $\|x(t)\| > \gamma_1$. Since $\|x(t)\| \leq \gamma_0$ for all $t \in \mathbb{Z}_+$, invoking the inequality in Eq(15) yields that

$$W(t+1) = w_1^\top x(t+1) = w_1^\top F(h, x(t)) \geq w_1^\top A_{\gamma_0} x(t) \geq c_1\|x(t)\| \geq c_1\gamma_1 =: \rho_2\,,$$

for some suitable positive constant c_1, the existence of which follows as $w_1^\top A_{\gamma_0} \gg 0$.

The conjunction of the above two cases yields that

$$W(t+1) \geq \min\{\rho_1 W(t), \rho_2\}, \quad \forall\, t \in \mathbb{Z}_+, \quad W(0) = w_1^\top x(0) > 0\,.$$

A consequence of $\rho_1 > 1$ is that the difference equation

$$Z(t+1) = \min\{\rho_1 Z(t), \rho_2\}, \quad \forall\, t \in \mathbb{Z}_+, \quad Z(0) = w_1^\top x(0) > 0\,,$$

converges to $\rho_2 > 0$ in finite time. In fact, setting $\xi := \min_{z\in\Gamma} w_1^\top z$, and assuming that $\xi < \rho_2$, it follows that $Z(t + \ell') \geq \rho_2$ for all $t \in \mathbb{Z}_+$ and all $x(0) \in \Gamma$ once $\ell' \in \mathbb{N}$ is such that

$$\rho_1^{\ell'}\xi \geq \rho_2, \quad \text{that is,} \quad \ell' = \text{floor}\Big(\log_{\rho_1}(\rho_2/\xi)\Big) + 1\,, \tag{1.4}$$

(omitting the $+1$ above if the $\log_{\rho_1}$ term is itself an integer). The function

$$\mathbb{R}_+ \to \mathbb{R}_+, \quad z \mapsto \min\{\rho_1 z, \rho_2\}\,,$$

is non-decreasing, and so the comparison principle for difference equations yields that

$$W(t) \geq Z(t) \quad \forall\, t \in \mathbb{Z}_+\,.$$

(In the case of difference equations, this result is readily established by induction. For comparison principles for ordinary differential equations, we refer the reader to, for example, [2, Chapter II].) Letting $c_2 > 0$ denote a norm equivalence constant such that $W(t) \leq c_2\|x(t)\|$, it follows that $\varepsilon = \rho_2/c_2$ is a persistence threshold for the difference equation in Eq(13). Observe that ε is independent of Γ. Putting the above conclusions together, we see that Eq(13) is uniformly ultimately semi-globally id-persistent.

To verify the affine-linear growth property of ℓ', let $0 < \delta < \Delta$ and $\gamma \in (0,1)$ be fixed as in Eq(11). For $t \in \mathbb{Z}_+$, we estimate that

$$\xi_t := \min_{z\in\Gamma_\gamma(t)} w_1^\top z \geq c_3' \min_{z\in\Gamma_\gamma(t)} \|z\| = c_3'\delta\gamma^t = c_3\gamma^t\,,$$

for some positive constants c_3', c_3. To bound ℓ_t' we substitute this value of ξ_t into Eq(1.4) and invoke elementary properties of logarithms to yield

$$\begin{aligned}\ell_t' &= \text{floor}\Big(\log_{\rho_1}(\rho_2/\xi)\Big) + 1 \leq \log_{\rho_1}(\rho_2/\xi) + 1 \leq \log_{\rho_1}\big((\rho_2/c_3)(\gamma^{-1})^t\big) + 1 \\ &= t\log_{\rho_1}(\gamma^{-1}) + \log_{\rho_1}(\rho_2/c_3) + 1, \quad \forall\, t \in \mathbb{Z}_+ .\end{aligned}$$

We conclude that ℓ_t' is affinely-linearly bounded.

Finally, the inequality in Eq(1.3) holds with $d = \gamma_0$, and yields constant $c > 0$ and integer $\nu \in \mathbb{Z}_+$ such that

$$\|y(t+\ell+\nu)\| \geq c\|x(t+\ell)\| \geq c\varepsilon, \quad \forall\, t \in \mathbb{Z}_+, \quad \forall\, x(0) \in \Gamma .$$

We conclude that Eq(13) is uniformly ultimately semi-globally η-persistent. Moreover, since $\ell_t = \ell_t' + \nu$, it follows that ℓ_t is also affinely-linearly bounded and, hence, hypothesis **(H3)** holds. The proof is complete. □

2 Derivation of hypotheses for the Pielou model

Again, we note that here, we use Eq(x) to refer to equations from the main text, where x is the equation number as it appears in the main text. Whereas, when referring to equations that appear here, but not in the main text, we use Eq(2.y), where y refers to the equation number within this section of S1 Appendix.

We verify properties **(H1)**–**(H4)** for the Pielou model in Eq(41). Since the functions $\mathbb{R}_+ \to \mathbb{R}_+$ given by

$$z \mapsto \max_h \frac{a_h z}{c_h + z} \quad \text{and} \quad z \mapsto \max_h \frac{b_h z}{d_h + z} ,$$

are bounded, the right-hand side of Eq(41) is bounded, uniformly in h. Therefore, it follows that **(H1)** holds (note, with bounds which are independent of $\Gamma \subseteq \mathbb{R}_+^2$ and $R > 0$ as well).

For hypothesis **(H2)**, let $\gamma_1 > 0$ be given. We estimate that, for all $h \in \underline{q}$, and all $z = (z_1, z_2) \in \mathbb{R}_+^2$ with $\|z\| \leq \gamma_1$,

$$F_1(h, z_1, z_2) = \frac{a_h z_2}{c_h + z_2} \mathrm{e}^{-z_1} \geq \Big(\min_h \frac{a_h}{c_h + \gamma_1} \mathrm{e}^{-\gamma_1}\Big) z_2 ,$$

and

$$F_2(h, z_1, z_2) = \frac{b_h z_1}{d_h + z_1} \mathrm{e}^{-z_2} \geq \Big(\min_h \frac{b_h}{d_h + \gamma_1} \mathrm{e}^{-\gamma_1}\Big) z_1 .$$

We conclude that there exists $\delta > 0$ such that

$$F\Big(h, \begin{pmatrix} z_1 \\ z_2 \end{pmatrix}\Big) \geq \delta \begin{pmatrix} z_2 \\ z_1 \end{pmatrix}, \quad \forall\, h \in \underline{q},\ \forall\, z = \begin{pmatrix} z_1 \\ z_2 \end{pmatrix} \in \mathbb{R}_+^2,\ \|z\| \leq \gamma_1 ,$$

so that

$$\|F(h,z)\| \geq \delta\|z\|, \quad \forall\, h \in \underline{q},\ \forall\, z = \begin{pmatrix} z_1 \\ z_2 \end{pmatrix} \in \mathbb{R}_+^2,\ \|z\| \leq \gamma_1 , \tag{2.1}$$

from which property **(H2)** follows and, moreover, so does **(H4)** since $y = x$.

We claim that the conditions $a_h/c_h > 1$ and $b_h/d_h > 1$ for fixed strategy $h \in \underline{q}$ are sufficient for hypothesis **(H3)** to hold. Note that these conditions are satisfied by strategy 1, see Table 5 in the main text. To establish the claim, fix such h and let sufficiently small $\gamma_2 > 0$ and $\rho > 1$ satisfy

$$\min\Big\{ \frac{a_h}{c_h + \gamma_2} \mathrm{e}^{-\gamma_2}, \frac{b_h}{d_h + \gamma_2} \mathrm{e}^{-\gamma_2} \Big\} > \rho ,$$

the existence of $\gamma_2 > 0$ following by continuity.

Therefore, for all $z = (z_1, z_2) \in \mathbb{R}_+^2$ with $\|z\| \leq \gamma_2$, it follows that

$$F_1(h, z_1, z_2) = \frac{a_h z_2}{c_h + z_2} \mathrm{e}^{-z_1} \geq \Big(\frac{a_h}{c_h + \gamma_2} \mathrm{e}^{-\gamma_2}\Big) z_2 \geq \rho z_2 ,$$

and

$$F_2(h, z_1, z_2) = \frac{b_h z_1}{d_h + z_1} \mathrm{e}^{-z_2} \geq \Big(\frac{b_h}{d_h + \gamma_2} \mathrm{e}^{-\gamma_2}\Big) z_1 \geq \rho z_1 .$$

As in the proof of property **(H2)**, we conclude that Eq(2.1) holds with δ and γ_1 replaced by $\rho > 1$ and γ_2, respectively. The proof of statement (iv) of Lemma 2 with $W(t)$ there replaced by $\|x(t)\|$ starting at the exhaustive cases now applies, and yields that property **(H3)** holds, as required.

3 Extra material for the final section of our simulation results

Here, Section 3.1 provides the data used to plot Figures 6 and 7 in the main text. Sections 3.2 and 3.3 include boxplots and data tables when the 24 switching systems are organised by what update law is used and what additional changes are added, respectively. Section 3.4 includes boxplots and data tables when the 24 switching systems are organised by which combination of update law and additional changes are applied. Sections 3.2 and 3.3 split the switching systems up into three categories whilst Section 3.4 splits the switching systems up into eight categories.

3.1 Data from the figures in the paper

Table 3.1 contains the data to produce Figure 10 in the main text. Tables 3.2, 3.3 and 3.4 contain the data to produce Figures 11A, 11B and 11C, respectively.

Switching System	Percentage bound in Strategy 1	Percentage bound in Strategy 2	Percentage bound in Strategy 3
1	0	23	77
2	0	24	76
3	0	100	0
4	0	23	77
5	0	100	0
6	0	24	76
7	0	100	0
8	0	100	0
9	0	20	80
10	0	21	79
11	0	100	0
12	0	20	80
13	0	100	0
14	0	21	79
15	0	100	0
16	0	100	0
17	0	69	31
18	0	68	32
19	0	100	0
20	0	69	31
21	0	100	0
22	0	68	32
23	0	100	0
24	0	100	0

Table 3.1: Percentage of the initial condition runs that converge in each strategy. See Figure 10 in the main text.

Switching System	Median	25th percentile	75th percentile	Lower adjacent	Upper adjacent	Minimum	Maximum	Outliers
1	31	26	35	13	41	6	96	20
2	30	26	35	13	46	6	87	20
3	53	47	59.5	32	67	6	67	17
4	31	26	34.5	14	41	6	96	21
5	52	47	59	30	67	6	67	14
6	30	26	34	14	41	6	87	22
7	54	47.5	59.5	32	67	6	67	15
8	52	47	59.5	30	67	6	67	15
9	15	11	19	6	31	6	103	14
10	15	10	21.5	6	31	6	93	14
11	55	49	61.5	34	69	8	69	18
12	14.5	11	18.5	6	29	6	103	13
13	54	49	60.5	32	69	8	69	16
14	14	10	19	6	31	6	93	11
15	55	49.5	61.5	34	69	8	69	16
16	54	49	60.5	32	69	8	69	17
17	12	7	25	2	49	2	59	1
18	12	7	25	2	45	2	58	2
19	12	7	34.5	2	45	2	45	0
20	12	7	25	2	49	2	59	1
21	12	7	35	2	47	2	47	0
22	12	7	25	2	39	2	55	1
23	12	7	34	2	45	2	45	0
24	12	7	35	2	48	2	48	0

Table 3.2: Box plot data for the time taken for s to converge by switching system type. See Figure 11A in the main text.

Switching System	Median	25th percentile	75th percentile	Lower adjacent	Upper adjacent	Minimum	Maximum	Outliers
1	4.26	3.31	4.98	1.58	6.45	1.58	6.45	0
2	4.26	3.32	4.98	1.89	6.45	1.89	6.45	0
3	3.57	3.41	3.91	2.70	4.58	2.70	6.45	6
4	4.26	3.31	4.98	1.58	6.45	1.58	6.45	0
5	3.58	3.41	3.91	2.70	4.58	2.70	6.45	6
6	4.26	3.36	4.98	1.89	6.45	1.89	6.45	0
7	3.57	3.41	3.91	2.70	4.58	2.70	6.45	6
8	3.58	3.41	3.91	2.70	4.58	2.70	6.45	6
9	4.35	3.39	4.98	1.47	6.45	1.47	6.45	0
10	4.35	3.39	4.98	1.78	6.45	1.78	6.45	0
11	3.56	3.42	3.91	2.70	4.58	2.70	6.45	6
12	4.35	3.39	4.98	1.47	6.45	1.47	6.45	0
13	3.56	3.42	3.91	2.70	4.58	2.70	6.45	6
14	4.35	3.42	4.98	1.78	6.45	1.78	6.45	0
15	3.56	3.42	3.91	2.70	4.58	2.70	6.45	6
16	3.56	3.42	3.91	2.70	4.58	2.70	6.45	6
17	4.08	3.53	4.98	2.09	6.45	2.09	6.45	0
18	4.08	3.53	4.98	2.46	6.45	2.46	6.45	0
19	3.91	3.45	4.47	2.70	5.69	2.70	6.45	3
20	4.08	3.53	4.98	2.09	6.45	2.09	6.45	0
21	3.91	3.45	4.47	2.70	5.69	2.70	6.45	3
22	4.08	3.53	4.98	2.46	6.45	2.46	6.45	0
23	3.91	3.45	4.47	2.70	5.69	2.70	6.45	3
24	3.91	3.45	4.47	2.70	5.69	2.70	6.45	3

Table 3.3: Box plot data for the minimum observed population size (to 2dp) reached before s converged, by switching system type. See Figure 11B in the main text.

Switching System	Median	25th percentile	75th percentile	Lower adjacent	Upper adjacent	Minimum	Maximum	Outliers
1	2	1	2	0	3	0	4	2
2	2	1	2	0	3	0	4	1
3	4	3	4	2	4	0	4	8
4	2	1	2	0	3	0	4	2
5	4	3	4	2	4	0	4	8
6	2	1	2	0	3	0	4	1
7	4	3	4	2	5	0	5	8
8	4	3	4	2	5	0	5	8
9	2	1	2	0	3	0	4	2
10	2	1	2	0	3	0	4	1
11	4	3	4	2	4	0	4	7
12	2	1	2	0	3	0	4	2
13	4	3	4	2	4	0	4	7
14	2	1	2	0	3	0	4	1
15	4	3	4	2	5	0	5	7
16	4	3	4	2	5	0	5	7
17	1	1	1	1	1	0	2	35
18	1	1	1	1	1	0	3	37
19	1	1	2	0	3	0	4	8
20	1	1	1	1	1	0	2	35
21	1	1	3	0	4	0	4	0
22	1	1	1	1	1	0	2	37
23	1	1	2	0	3	0	4	8
24	1	1	2	0	3	0	5	10

Table 3.4: Box plot data for the number of times switched strategy before s converged, by switching system type. See Figure 11C in the main text.

3.2 Data organised by update law

We compare the switching systems based on which update law is applied and provide the corresponding box plots across Figures 3.1a, 3.1b and 3.1c. The data is provided in Tables 3.5, 3.6 and 3.7.

Switching System	Median	25th percentile	75th percentile	Lower adjacent	Upper adjacent	Minimum	Maximum	Outliers
Original	37	28	55	6	93	6	96	2
MA	28	14	57	6	103	6	103	0
RT	12	7	26	2	49	2	59	5

Table 3.5: Box plot data for the time taken for s to converge, by update law. See Figure 3.1a.

Switching System	Median	25th percentile	75th percentile	Lower adjacent	Upper adjacent	Minimum	Maximum	Outliers
Original	3.73	3.40	4.50	1.76	6.07	1.58	6.45	24
MA	3.38	3.42	4.56	1.76	6.17	1.47	6.45	18
RT	3.93	3.48	4.78	2.09	6.45	2.09	6.45	0

Table 3.6: Box plot data for the minimum observed population size (to 2dp) reached before s converged, by update law. See Figure 3.1b.

Switching System	Median	25th percentile	75th percentile	Lower adjacent	Upper adjacent	Minimum	Maximum	Outliers
Original	2	2	4	0	5	0	5	0
MA	2	2	4	0	5	0	5	0
RT	1	1	1	1	1	0	5	348

Table 3.7: Box plot data for the number of times switched strategy before s converged, by update law. See Figure 3.1c.

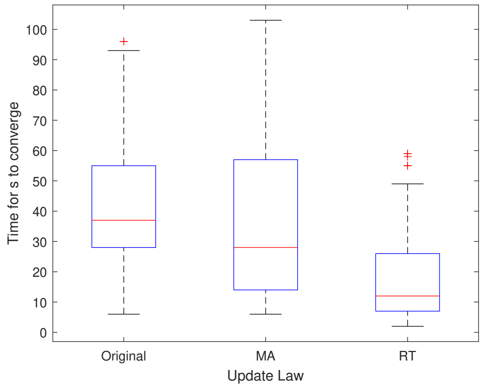

(a) Time taken for s to converge by update law. Summary statistics are recorded in Table 3.5.

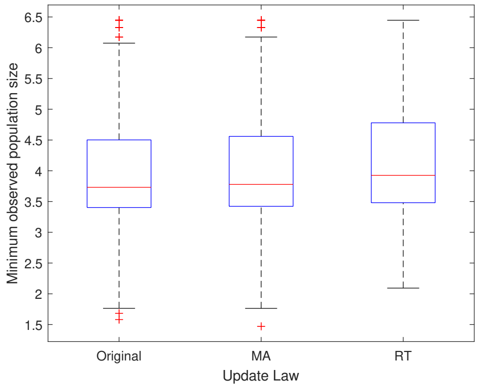

(b) Minimum observed population size reached before s converged, by update law. Summary statistics are recorded in Table 3.6.

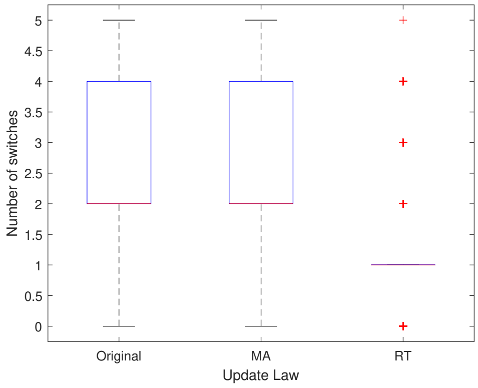

(c) Number of times switching system switched strategy before s converged, by update law. Summary statistics are recorded in Table 3.7.

Figure 3.1: Box plot for the switching systems detailed in the final section of simulation results, where the data is organised into groups associated with the update law used. For each switching system, 100 sets of initial conditions for x_0, s_0, and $\tau(1)$ are tested.

3.3 Data organised by additional changes added to the switching system

We compare the switching systems based on which additional changes are applied and provide the corresponding box plots across Figures 3.2a, 3.2b and 3.2c. The data is provided in Tables 3.8, 3.9 and 3.10.

Switching System	Median	25th percentile	75th percentile	Lower adjacent	Upper adjacent	Minimum	Maximum	Outliers
MST	28	12	50	2	93	2	93	0
OR	48	18	57	2	69	2	69	0
DRS	27	12	51	2	103	2	103	0

Table 3.8: Box plot data for the time taken for s to converge, by additional changes added. See Figure 3.2a.

Switching System	Median	25th percentile	75th percentile	Lower adjacent	Upper adjacent	Minimum	Maximum	Outliers
MST	3.87	3.42	4.60	1.78	6.33	1.78	6.45	12
OR	3.65	2.70	3.93	2.70	4.67	2.70	6.45	128
DRS	3.87	3.42	4.60	1.68	6.33	1.47	6.45	14

Table 3.9: Box plot data for the minimum observed population size (to 2dp) reached before s converged, by additional changes added. See Figure 3.2b.

Switching System	Median	25th percentile	75th percentile	Lower adjacent	Upper adjacent	Minimum	Maximum	Outliers
MST	2	1	3	0	5	0	5	0
OR	3	1	4	0	5	0	5	0
DRS	2	1	3	0	5	0	5	0

Table 3.10: Box plot data for the number of times switched strategy before s converged, by additional changes added. See Figure 3.2c.

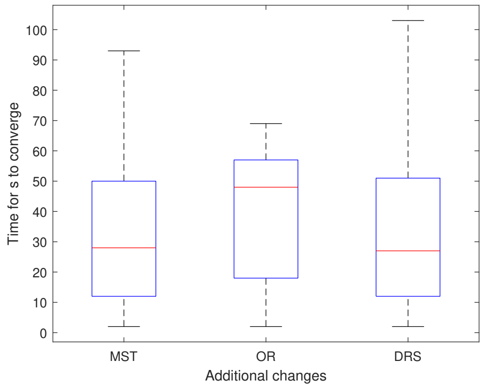

(a) Time taken for s to converge by additional changes added to the switching system. Summary statistics are recorded in Table 3.8.

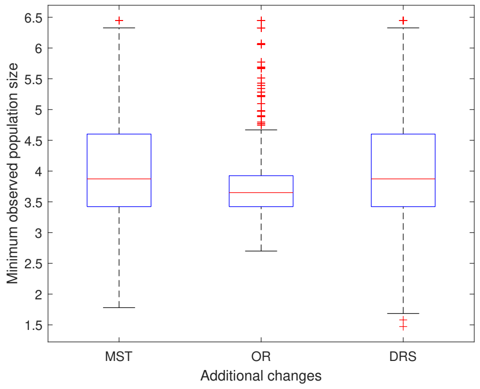

(b) Minimum observed population size reached before s converged, by additional changes added to the switching system. Summary statistics are recorded in Table 3.9.

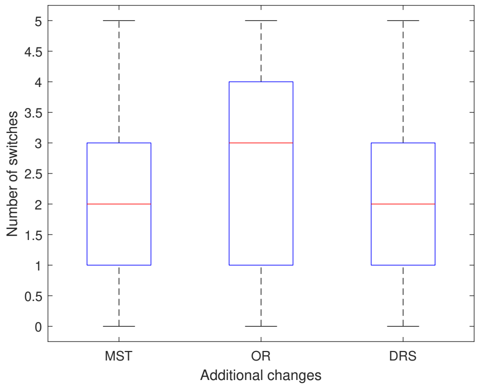

(c) Number of times switching system switched strategy before s converged, by additional changes added to the switching system. Summary statistics are recorded in Table 3.10.

Figure 3.2: Box plot for the switching systems detailed in the final section of our simulation results, where the data is organised into groups associated which the additional changes added to the switching system. For each switching system, 100 sets of initial conditions for x_0, s_0, and $\tau(1)$ are tested.

3.4 Data organised by the combination of additional changes added to the switching system

We compare the switching systems based on which combination of additional changes is applied and provide the corresponding box plots across Figures 3.3a, 3.3b and 3.3c. The data is provided in Tables 3.11, 3.12 and 3.13.

Switching System	Median	25th percentile	75th percentile	Lower adjacent	Upper adjacent	Minimum	Maximum	Outliers
None	18	11	31	2	61	2	103	22
MST	20.5	10.5	31	2	60	2	93	19
OR	48	18	57	2	69	2	69	0
DRS	17.5	11	30	2	49	2	103	23
MST&OR	48	19.5	56	2	69	2	69	0
MST&DRS	18	10	29.5	2	56	2	93	19
OR&DRS	48	18	57	2	69	2	69	0
MST&OR&DRS	48	17.5	56	2	69	2	69	0

Table 3.11: Box plot data for the time taken for s to converge, by the combination of additional changes added. See Figure 3.3a.

Switching System	Median	25th percentile	75th percentile	Lower adjacent	Upper adjacent	Minimum	Maximum	Outliers
None	4.21	3.42	4.98	1.47	6.45	1.47	6.45	0
MST	4.21	3.42	4.98	1.78	6.45	1.78	6.45	0
OR	3.65	3.42	3.93	2.70	4.67	2.70	6.45	32
DRS	4.21	3.42	4.98	1.47	6.45	1.47	6.45	0
MST&OR	3.65	3.42	3.93	2.70	4.67	2.70	6.45	32
MST&DRS	4.21	3.43	4.98	1.78	6.45	1.78	6.45	0
OR&DRS	3.65	3.42	3.93	2.70	4.67	2.70	6.45	32
MST&OR&DRS	3.65	3.42	3.93	2.70	4.67	2.70	6.45	32

Table 3.12: Box plot data for the minimum observed population size (to 2dp) reached before s converged, by the combination of additional changes added. See Figure 3.3b.

Switching System	Median	25th percentile	75th percentile	Lower adjacent	Upper adjacent	Minimum	Maximum	Outliers
None	1	1	2	0	3	0	4	4
MST	1	1	2	0	3	0	4	2
OR	3	1	4	0	4	0	4	0
DRS	1	1	2	0	3	0	4	4
MST&OR	3	1	4	0	4	0	4	0
MST&DRS	1	1	2	0	3	0	4	2
OR&DRS	3	1	4	0	5	0	5	0
MST&OR&DRS	3	1	4	0	5	0	5	0

Table 3.13: Box plot data for the number of times switched strategy before s converged, by the combination of additional changes added. See Figure 3.3c.

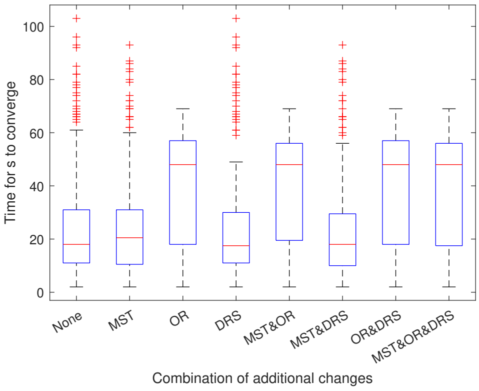

(a) Time taken for s to converge by the combination of additional changes added. Summary statistics are recorded in Table 3.11.

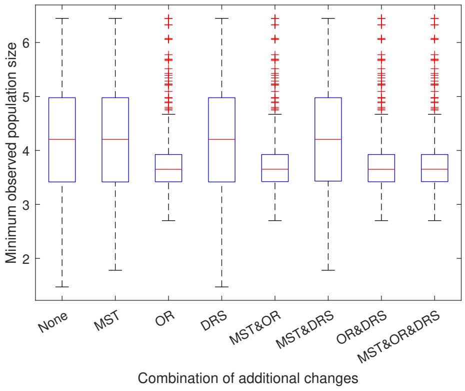

(b) Minimum observed population size reached before s converged, by the combination of additional changes added. Summary statistics are recorded in Table 3.12.

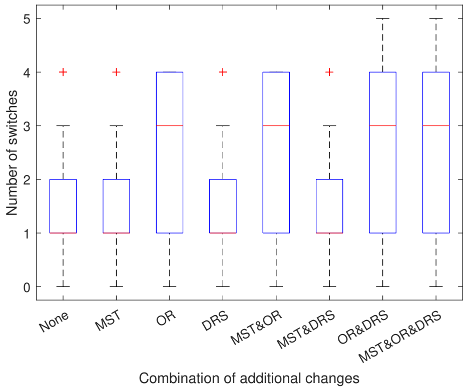

(c) Number of times switching system switched strategy before s converged, by the combination of additional changes added. Summary statistics are recorded in Table 3.13.

Figure 3.3: Box plot for the switching systems detailed in the final section of our simulation results, where the data is organised into groups associated with which combination of additional changes is added. For each switching system, 100 sets of initial conditions for x_0, s_0, and $\tau(1)$ are tested.

References

1. Berman A, Plemmons RJ. Nonnegative matrices in the mathematical sciences. Philadelphia, PA: SIAM; 1994.

2. Walter W. Differential and integral inequalities. vol. 55 of Ergebnisse der Mathematik und ihrer Grenzgebiete. Heidelberg: Springer-Verlag Berlin; 1970.
